# International society of sports nutrition position stand: caffeine and exercise performance

**DOI:** 10.1186/s12970-020-00383-4

**Published:** 2021-01-02

**Authors:** Nanci S. Guest, Trisha A. VanDusseldorp, Michael T. Nelson, Jozo Grgic, Brad J. Schoenfeld, Nathaniel D. M. Jenkins, Shawn M. Arent, Jose Antonio, Jeffrey R. Stout, Eric T. Trexler, Abbie E. Smith-Ryan, Erica R. Goldstein, Douglas S. Kalman, Bill I. Campbell

**Affiliations:** 1 Department of Nutritional Sciences, Faculty of Medicine, University of Toronto, 1 King’s College Circle, Room 5326A, Toronto, ON M5S 1A8 Canada; 2 Department of Exercise Science and Sport Management, Kennesaw State University, Kennesaw, GA 30144 USA; 3 Carrick Institute, Cape Canaveral, FL 32920 USA; 4 Institute for Health and Sport (IHES), Victoria University, Melbourne, Australia; 5 Department of Health Sciences, CUNY Lehman College, Bronx, NY 10468 USA; 6 Department of Health and Human Physiology, University of Iowa, Iowa City, IA 52240 USA; 7 Department of Exercise Science, Arnold School of Public Health, University of South Carolina, Colombia, SC 29208 USA; 8 School of Social and Health Sciences, Leeds Trinity University, Leeds, UK; 9 Exercise and Sport Science, Nova Southeastern University, Davie, FL 33314 USA; 10 Institue of Exercise Physiology and Rehabilitation Science, University of Central Florida, Orlando, FL 32816 USA; 11 Stronger by Science LLC, Raleigh, NC USA; 12 Department of Exercise and Sport Science, Applied Physiology Laboratory, University of North Carolina, Chapel Hill, NC 27599 USA; 13 Nutrion Department, College of Osteopathic Medicine, Nova Southeastern University, Fort Lauderdale, FL 33314 USA; 14 Scientific Affairs. Nutrasource, Guelph, ON Canada; 15 Performance & Physique Enhancement Laboratory, University of South Florida, Tampa, FL 33612 USA

## Abstract

Following critical evaluation of the available literature to date, The International Society of Sports Nutrition (ISSN) position regarding caffeine intake is as follows:

1. Supplementation with caffeine has been shown to acutely enhance various aspects of exercise performance in many but not all studies. Small to moderate benefits of caffeine use include, but are not limited to: muscular endurance, movement velocity and muscular strength, sprinting, jumping, and throwing performance, as well as a wide range of aerobic and anaerobic sport-specific actions.
2. Aerobic endurance appears to be the form of exercise with the most consistent moderate-to-large benefits from caffeine use, although the magnitude of its effects differs between individuals.
3. Caffeine has consistently been shown to improve exercise performance when consumed in doses of 3–6 mg/kg body mass. Minimal effective doses of caffeine currently remain unclear but they may be as low as 2 mg/kg body mass. Very high doses of caffeine (e.g. 9 mg/kg) are associated with a high incidence of side-effects and do not seem to be required to elicit an ergogenic effect.
4. The most commonly used timing of caffeine supplementation is 60 min pre-exercise. Optimal timing of caffeine ingestion likely depends on the source of caffeine. For example, as compared to caffeine capsules, caffeine chewing gums may require a shorter waiting time from consumption to the start of the exercise session.
5. Caffeine appears to improve physical performance in both trained and untrained individuals.
6. Inter-individual differences in sport and exercise performance as well as adverse effects on sleep or feelings of anxiety following caffeine ingestion may be attributed to genetic variation associated with caffeine metabolism, and physical and psychological response. Other factors such as habitual caffeine intake also may play a role in between-individual response variation.
7. Caffeine has been shown to be ergogenic for cognitive function, including attention and vigilance, in most individuals.
8. Caffeine may improve cognitive and physical performance in some individuals under conditions of sleep deprivation.
9. The use of caffeine in conjunction with endurance exercise in the heat and at altitude is well supported when dosages range from 3 to 6 mg/kg and 4–6 mg/kg, respectively.
10. Alternative sources of caffeine such as caffeinated chewing gum, mouth rinses, energy gels and chews have been shown to improve performance, primarily in aerobic exercise.
11. Energy drinks and pre-workout supplements containing caffeine have been demonstrated to enhance both anaerobic and aerobic performance.

## Introduction

Caffeine is the world’s most widely consumed psychoactive substance and naturally occurs in dozens of plant species, including coffee, tea and cocoa. Caffeine is ingested most frequently in the form of a beverage such as coffee, soft drinks and tea, although the consumption of many functional beverages, such as energy drinks, has been on a steady rise in the past two decades [1]. In Western countries, approximately 90% of adults consume caffeine on a regular basis, with dietary caffeine consumption of U.S. adult men and women estimated at approximately 200 mg/day in a 2009–2010 survey [2–4]. In young adults and exercising individuals, there has also been a rise in the consumption of other caffeine-containing products, including energy drinks [1, 3], ‘pre-workout supplements’, chewing gum, energy gels and chews, aerosols, and many other novel caffeinated food products [5]. Caffeine-containing products have a range of doses per serving, from 1 mg in milk chocolate up to > 300 mg in some dietary supplements [6].

Caffeine and its effects on health have been a longstanding topic of interest, and caffeine continues to be a dietary compound of concern in public health, as indicated by extensive investigations [7–10]. At the same time, caffeine has become ubiquitous in the sporting world, where there is keen interest in better understanding the impact of caffeine on various types of exercise performance. Accordingly, caffeine has dominated the ergogenic aids and sport supplement research domain over the past several decades [11–13].

## Caffeine in sport: a brief history

In the early days (1900s) of modern sport, concoctions of plant-based stimulants, including caffeine and other compounds such as cocaine, strychnine, ether, heroin and nitroglycerin, were developed secretly by trainers, athletes and coaches, in what appears to be evidence for early day ergogenic aids designed to provide a competitive advantage [14]. The use of various pharmaceutical cocktails by endurance athletes continued until heroin and cocaine became restricted to prescriptions in the 1920s, and further when the International Olympic Committee (IOC) introduced anti-doping programs in the late 1960s [15].

Some of the earliest published studies on caffeine came from two psychologists and colleagues William Rivers and Harald Webber, at Cambridge University, who both had an interest in disentangling the psychological and physiological effects of substances like caffeine and alcohol. Rivers and Webber, using themselves as subjects, investigated the effects of caffeine on muscle fatigue. The remarkable well-designed studies carried out from 1906 to 1907 used double-blinded placebo-controlled trials and standardization for diet (i.e. caffeine, alcohol), and were described in a 1907 paper in the *Journal of Physiology* [16]. Significant research on the effects of caffeine on exercise performance with more subjects, different sports, and exploring variables such as the effects between trained and untrained individuals, began and continued through the 1940s [14, 17]. However, it was the series of studies investigating the benefits of caffeine in endurance sports in the Human Performance Laboratory at Ball State University in the late 1970s, led by David Costill [18, 19] and others [20], that sparked a generation of research on the effects of caffeine in exercise metabolism and sports performance.

## Caffeine sources

Along with naturally occurring sources, such as coffee, tea and cocoa, caffeine is also added to many foods, beverages and novelty products, such as jerky, peanut butter, and candy, in both synthetic (e.g. powder) and natural (e.g. guarana, kola nut) forms. Synthetic caffeine is also an ingredient in several over-the-counter and prescription medications, as it is often used in combination with analgesic and diuretic drugs to amplify their pharmacological potency [21].

Approximately 96% of caffeine consumption from beverages comes from coffee, soft drinks and tea [22]. Additionally, there are varying levels of caffeine in the beans, leaves and fruit of more than 60 plants, resulting in great interest in herbal and other plant-based supplements [23–26]. Caffeine-containing energy drink consumption [27–31] and co-ingestion of caffeine with (e.g. “pre-workouts”), or in addition to, other supplements (e.g. caffeine + creatine) is also popular among exercising individuals [32–39]. To date, the preponderance of caffeine and exercise performance literature has utilized anhydrous caffeine (in a capsule) [40–46] for simpler dose standardization and placebo creation. There is also a growing body of literature studying the effects of using alternate delivery methods of caffeine during exercise [5] such as coffee [18, 47–56], energy drinks, herbal formulas [57] and ‘pre-workout’ formulas, among others. A review of alternate caffeine forms may be found in the *Alternative caffeine sources* section and Tables 4, 5, 6, 7 and 8.

## Caffeine legality in sport

Anti-doping rules apply to most sports, especially in those where athletes are competing at national and international levels. The IOC continues to recognize that caffeine is frequently used by athletes because of its reported performance-enhancing or ergogenic effects [109]. Caffeine was added to the list of banned substances by the IOC in 1984 and the World Anti-Doping Agency (WADA) in 2000. A doping offense was defined as having urinary caffeine concentrations exceeding a cut-off of 15 μg/ml. In 1985, the threshold was reduced to 12 μg/ml [110]. The cut-off value was chosen to exclude typical amounts ingested as part of common dietary or social coffee drinking patterns, and to differentiate it from what was considered to be an aberrant use of caffeine for the purpose of sports performance enhancement [111].

The IOC and WADA removed the classification of caffeine as a “controlled” substance in 2004, leading to a renewed interest in the use of caffeine by athletes. However, caffeine is still monitored by WADA, and athletes are encouraged to maintain a urine caffeine concentration below the limit of 12 μg/ml urine which corresponds to 10 mg/kg body mass orally ingested over several hours, and which is more than triple the intake reported to enhance performance [112, 113]. Interestingly, caffeine is also categorized as a banned substance by the National Collegiate Athletic Association (NCAA), if urinary caffeine concentration exceeds 15 μg/ml, which is greater than the “monitored substance” level set for WADA [114], and also well above amounts that are deemed ergogenic.

A comparison of caffeine concentrations obtained during in-competition doping control from athletes in several sports federations pre− 2004 versus post-2004, indicated that average caffeine concentrations decreased in 2004 after removal from the prohibited substance list [110]. Reports on over 20,000 urine samples collected and analyzed after official national and international competitions between 2004 and 2008, and again in 2015 using 7500 urine samples found overall prevalence of caffeine use across various sports to be about 74% in the 2004 to 2008 time period and roughly 76% in 2015. The highest use of caffeine was among endurance athletes in both studies [115, 116]. Urinary caffeine concentration significantly increased from 2004 to 2015 in athletics, aquatics, rowing, boxing, judo, football, and weightlifting; however, the sports with the highest urine caffeine concentration in 2015 were cycling, athletics, and rowing [116].

## Caffeine pharmacokinetics

Caffeine or 1,3,7-trimethylxanthine, is an odorless white powder that is soluble in both water and lipids and has a bitter taste. It is rapidly absorbed from the gastrointestinal tract, mainly from the small intestine but also in the stomach [117]. In saliva, caffeine concentration reaches 65–85% of plasma levels, and is often used to non-invasively monitor compliance for ingestion or abstinence of caffeine [118]. Caffeine is effectively distributed throughout the body by virtue of being sufficiently hydrophobic to allow easy passage through most, if not all biological membranes, including the blood-brain barrier [119]. When caffeine is consumed it appears in the blood within minutes, with peak caffeine plasma concentrations after oral administration reported to occur at times (T_max_) ranging from 30 to 120 min [43, 120–122]. The absolute bioavailability of caffeine is very high and reaches near 100% as seen in studies reporting areas under the plasma concentration-time curves (AUC) [120]. Once caffeine is absorbed, there appears to be no hepatic first-pass effect (i.e.*,* the liver does not appear to remove caffeine as it passes from the gut to the general circulation), as evidenced by similar plasma concentration curves when administered by either oral or intravenous routes [123]. Caffeine absorption from food and beverages does not seem to be dependent on age, gender, genetics or disease, or the consumption of drugs, alcohol or nicotine. However, the rates of caffeine metabolism and breakdown appear to differ between individuals through both environmental and genetic influences [3, 124, 125].

Over 95% of caffeine is metabolized in the liver by the Cytochrome P450 1A2 (CYP1A2) enzyme, a member of the cytochrome P450 mixed-function oxidase system, which metabolizes and detoxifies xenobiotics in the body [126]. CYP1A2 catalyzes the demethylation of caffeine into the primary metabolites paraxanthine (1,7-dimethylxanthine), theobromine (3,7-dimethylxanthine) and theophylline (1,3-dimethylxanthine), which account for approximately 84, 12, and 4%, of total caffeine elimination, respectively [127, 128]. These three caffeine metabolites undergo further demethylations and oxidation to urates in the liver with about 3–5% remaining in caffeine form when excreted in the urine [129, 130]. While the average half-life (t_1/2_) of caffeine is generally reported to be between 4 and 6 h, it varies between individuals and even may range from 1.5 to 10 h in adults [120]. The wide range of variability in caffeine metabolism is due to several factors. The rate of caffeine metabolism may be inhibited or decreased with pregnancy or use of hormonal contraceptives [125], increased or induced by heavy caffeine use [131] cigarette smoking [132] or modified in either direction by certain dietary factors [133] and/or variation in the *CYP1A2* gene, which will be discussed later [125, 132–134].

Several studies have also shown that the form of caffeine or its vehicle for entry into the body can modify the pharmacokinetics [58, 81, 119, 122]. One small trial (*n* = 3) evaluated T_max_ for a variety of beverages that all included 160 mg of caffeine but in different volumes of solution, and reported that T_max_ occurs at 0.5, 0.5, and 2 h for coffee, tea and cola, respectively [135]. In another study involving seven participants, caffeine plasma concentrations peaked rapidly at 30 min for capsule form, whereas caffeine absorption from cola and chocolate was delayed and produced lower plasma concentrations that peaked at roughly 90–120 min after consumption. This study also did not control for volume of administered solution (capsules and chocolate ingested with 360 ml water and 800 ml cola) [122]. Liguori et al. [136] evaluated a 400 mg dose of caffeine in 13 subjects and reported salivary caffeine T_max_ values of 42, 39 and 67 min, for coffee, sugar-free cola and caffeine capsules, respectively. However, fluid volume was again not standardized (coffee – 12 oz., sugar-free cola – 24 oz., capsules – volume of administered fluid not reported). The impact of temperature or rate of ingestion of caffeine has also been investigated, amidst concerns that cold energy drinks might pose a danger when chugged quickly, compared to sipping hot coffee. One study [121] compared five conditions that included: slow ingestion (20 min) of hot coffee, and fast (2-min) or slow (20-min) ingestion for both cold coffee and energy drinks. Similar to other caffeine pharmacokinetic studies [122, 135], White et al. [121] reported that although the rate of consumption, temperature, and source (coffee vs. energy drink) may be associated with slight differences in pharmacokinetic activity, these differences are small.

Chewing gum formulations appear to alter pharmacokinetics, as much of the caffeine released from the gum through mastication can be absorbed via the buccal cavity, which is considered faster due to its extensive vascularization, especially for low molecular weight hydrophobic agents [137]. Kamimori et al. [58] compared the rate of absorption and relative caffeine bioavailability from chewing gum compared to a capsule form of caffeine. Although caffeine administered in the chewing gum formulation was absorbed at a significantly faster rate, the overall bioavailability was comparable to the capsuled 100 and 200 mg caffeine dose groups. These pharmacokinetic findings are useful for military and sport purposes, where there is a requirement for rapid and maintained stimulation over specific periods of time. Chewing gum may also be advantageous due to reduced digestive requirements, where absorption of caffeine in other forms (capsule, coffee etc.) may be hindered by diminished splanchnic blood flow during moderate to intense exercise. Finally, there is a growing prevalence of caffeinated nasal and mouth aerosols administered directly in the mouth, under the tongue or inspired may affect the brain more quickly through several proposed mechanisms [5], although there are only a few studies to date to support this claim. The administration of caffeine via aerosol into the oral cavity appears to produce a caffeine pharmacokinetic profile comparable to the administration of a caffeinated beverage [81]. Nasal and mouth aerosols will be discussed further in another section.

## Mechanism of Action (MOA)

Although the action of caffeine on the central nervous system (CNS) has been widely accepted as the primary mechanism by which caffeine alters performance, several mechanisms have been proposed to explain the ergogenic effects of caffeine, including increased myofibrillar calcium availability [138, 139], optimized exercise metabolism and substrate availability [45], as well as stimulation of the CNS [140–142]. One of the earlier proposed mechanisms associated with the ergogenic effects of caffeine stemmed from the observed adrenaline (epinephrine)-induced enhanced free-fatty acid (FFA) oxidation after caffeine ingestion and consequent glycogen sparing, resulting in improved endurance performance [18, 45, 143]. However, this substrate-availability hypothesis was challenged and eventually dismissed, where after several performance studies it became clear that the increased levels of FFAs appeared to be higher earlier in exercise when increased demand for fuel via fat oxidation would be expected [141, 144, 145]. Furthermore, this mechanism could not explain the ergogenic effects of caffeine in short duration, high-intensity exercise in which glycogen levels are not a limiting factor. Importantly, several studies employing a variety of exercise modalities and intensities failed to show a decrease in respiratory exchange ratio (RER) and/or changes in serum FFAs, which would be indicative of enhanced fat metabolism during exercise when only water was ingested [144, 146–148]. Ingestion of lower doses of caffeine (1–3 mg/kg of body mass), which do not result in significant physiological responses (i.e. RER, changes in blood lactate, glucose), also appear to deliver measurable ergogenic effects, offering strong support for the CNS as the origin of reported improvements [43, 149, 150]. As such, focus has shifted to the action of caffeine during exercise within the central and peripheral nervous systems, which could alter the rate of perceived exertion (RPE) [151–154], muscle pain [151, 155–157], and possibly the ability of skeletal muscle to generate force [151].

Caffeine does appear to have some direct effects on muscle which may contribute to its ergogenicity. The most likely pathway that caffeine may benefit muscle contraction is through calcium ion (Ca^2+^) mobilization, which facilitates force production by each motor unit [138, 139, 150, 158]. Fatigue caused by the gradual reduction of Ca^2+^ release may be attenuated after caffeine ingestion [139, 159]. Similarly, caffeine may work, in part, in the periphery through increased sodium/potassium (Na^+^/K^+^) pump activity to potentially enhance excitation-contraction coupling necessary for muscle contraction [160]. Caffeine appears to employ its effects at various locations in the body, but the most robust evidence suggests that the main target is the CNS, which is now widely accepted as the primary mechanism by which caffeine alters mental and physical performance [141]. Caffeine is believed to exert its effects on the CNS via the antagonism of adenosine receptors, leading to increases in neurotransmitter release, motor unit firing rates, and pain suppression [151, 155–157, 161]. There are four distinct adenosine receptors, A_1_, A_2A_, A_2B_ and A_3_, that have been cloned and characterized in several species [162]. Of these subtypes, A_1_ and A_2A,_ which are highly concentrated in the brain, appear to be the main targets of caffeine [163]. Adenosine is involved in numerous processes and pathways, and plays a crucial role as a homeostatic regulator and neuromodulator in the nervous system [164]. The major known effects of adenosine are to decrease the concentration of many CNS neurotransmitters, including serotonin, dopamine, acetylcholine, norepinephrine and glutamate [163–165]. Caffeine, which has a similar molecular structure to adenosine, binds to adenosine receptors after ingestion and therefore increases the concentration of these neurotransmitters [163, 165]. This results in positive effects on mood, vigilance, focus, and alertness in most, but not all, individuals [166, 167].

Researchers have also characterized aspects of adenosine A_2A_ receptor function related to cognitive processes [168] and motivation [169, 170]. In particular, several studies have focused on the functional significance of adenosine A_2A_ receptors and the interactions between adenosine and dopamine receptors, in relation to aspects of behavioral activation and effort-related processes [168–171]. The serotonin receptor 2A (5-HT2A) has also been shown to modulate dopamine release, through mechanisms involving regulation of either dopamine synthesis or dopaminergic neuron firing rate [172, 173]. Alterations in 5-HTR2A receptors may therefore affect dopamine release and upregulation of dopamine receptors [174, 175]. A possible mechanism for caffeine’s ergogenicity may involve variability in 5-HTR2A receptor activity, which may modulate dopamine release and consequently impact alertness, pain and motivation and effort [141]. 5-HTR2A receptors are encoded by the *HTR2A* gene, which serves as a primary target for serotonin signaling [176], and variations in the gene have been shown to affect 5-HTR2A receptor activity [177, 178]. This may therefore modulate dopamine activity, which may help to elucidate some of the relationships among neurotransmitters, genetic variation and caffeine response, and the subsequent impact on exercise performance.

Muscle pain has been shown to negatively affect motor unit recruitment and skeletal muscle force generation proportional to the subjective scores for pain intensity [179, 180]. In one study, progressively increased muscle pain intensity caused a gradual decrease in motor firing rates [179]. However, this decrease was not associated with a change in motor unit membrane properties demonstrating a central inhibitory motor control mechanism with effects correlated to nociceptive activity [179]. Other studies also indicate that muscle force inhibition by muscle pain is centrally mediated [181]. Accordingly, caffeine-mediated CNS mechanisms, such as dopamine release [182], are likely imputable for pain mitigation during high-intensity exercise [155–157, 181, 183–186]. Although there appears to be strong evidence supporting the analgesic effects of caffeine during intense exercise, others have found no effect [185, 187].

The attenuation of pain during exercise as a result of caffeine supplementation may also result in a decrease in the RPE during exercise. Two studies [183, 184] have reported that improvements in performance were accompanied by a decrease in pain perception as well as a decrease in RPE under caffeine conditions, but it is unclear which factor may have contributed to the ergogenic effect. Acute caffeine ingestion has been shown to alter RPE, where effort may be greater under caffeine conditions, yet it is not perceived as such [12, 152–154]. A meta-analysis [12] identified 21 studies using mostly healthy male subjects (74%) between the ages of 20 and 35 years and showed a 5.6% reduction in RPE during exercise following caffeine ingestion. An average improvement in performance of 11% was reported across all exercise modalities. This meta-analysis established that reductions in RPE explain up to 29% of the variance in the improvement in exercise performance [12]. Others have not found changes in RPE with caffeine use [187]. A more recent study by Green et al. [188] also showed that when subjects were instructed to cycle at specific RPE (effort) levels under caffeine conditions, the higher perceived intensity did not necessarily result in greater work and improved performance in all subjects equally. The authors noted that individual responses to caffeine might explain their unexpected findings.

In the last decade, our understanding of CNS fatigue has improved. Historically, it is well- documented that “psychological factors” can affect exercise performance and that dysfunction at any step in the continuum from the brain to the peripheral contractile machinery will result in muscular fatigue [189, 190]. The role of the CNS and its ‘motor drive’ effect was nicely shown by Davis et al. [191] who examined the effect of caffeine injected directly into the brains of rats on their ability to run to exhaustion on a treadmill. In this controlled study, rats were injected with either vehicle (placebo), caffeine, 5′-N-Ethylcarboxamido adenosine (NECA), an adenosine receptor agonist, or caffeine NECA together. Rats ran 80 min in the placebo trial, 120 min in the caffeine trial and only 25 min with NECA. When caffeine and NECA were given together, the effects appeared to cancel each other out, and run time was similar to placebo. When the study was repeated with peripheral intraperitoneal (body cavity) injections instead of brain injections, there was no effect on run performance. The authors concluded that caffeine increased running time by delaying fatigue through CNS effects, in part by blocking adenosine receptors [191]. Caffeine also appears to enhance cognitive performance more in fatigued than well-rested subjects [192–194]. This phenomenon is also apparent in exercise performance [195] both in the field [196] and in the lab [60, 63, 149].

### The placebo effect

The placebo effect is a beneficial outcome that cannot be attributed to a treatment or intervention but is brought about by the belief that one has received a positive intervention. For example, an individual may ingest a capsule with sugar or flour (a small amount of non-active ingredient) but believes that he/she ingested caffeine and experiences improvements in performance because of this belief [197]. The nocebo effect is directly opposite to this in that a negative outcome occurs following the administration of an intervention or lack of an intervention (e.g. knowingly ingesting a placebo) [198]. For example, the nocebo may be a substance without medical effects, but which worsens the health status of the person taking it by the negative beliefs and expectations of the patient. Similarly, the nocebo may be a ‘caffeine placebo’, where an individual’s performance is worse based on the belief that they did not ingest caffeine.

Several studies have provided evidence for placebo effects associated with caffeine ingestion [199–201] or other “beneficial” interventions [202] during exercise. An example of this was reported in a study [200] where well-trained cyclists exhibited a linear dose–response relationship in experimental trials from baseline to a moderate (4.5 mg/kg) and high dose (9 mg/kg) of caffeine respectively. Athletes improved as the perceived caffeine doses increased; however, a placebo was used in all interventions. Similarly, Saunders et al. [201] found that correct identification of caffeine appears to improve cycling performance to a greater extent than the overall effect of caffeine, where participants who correctly identified placebo showed possible harmful effects on performance. Therefore, readers are encouraged to consider whether studies that have explored the effects of caffeine on exercise have examined and reported the efficacy of the blinding of the participants.

## Caffeine and endurance exercise

Less than a 1% change in average speed is enough to affect medal rankings in intense Olympic endurance events lasting ~ 45 s to 8 min [203]. In other events, such as the men’s individual road race, the difference between the top three medalists was < 0.01% [204]. At the highest level of sports, competitors will be near their genetic potential, will have trained intensively, followed prudent recovery protocols, and will have exploited all strategies to improve their performance—the use of an ergogenic aid, when legal, safe and effective, is an alluring opportunity.

Caffeine has consistently been shown to improve endurance by 2–4% across dozens of studies using doses of 3–6 mg/kg body mass [13, 195, 205–207]. Accordingly, caffeine is one of the most prominent ergogenic aids and is used by athletes and active individuals in a wide variety of sports and activities involving aerobic endurance. Caffeine has been shown to benefit several endurance-type sports including cycling [60, 206, 208], running [91, 209, 210] cross-country skiing [211] and swimming [212].

Much of the caffeine-exercise body of literature has focused on endurance-type exercise, as this is the area in which caffeine supplementation appears to be more commonly used and likely beneficial in most, but not all, athletes [11–13]. For example, the caffeine concentration in over twenty thousand urine samples obtained for doping control from 2004 to 2008 was measured after official national and international competitions [110, 115]. The investigations concluded that roughly 74% of elite athletes used caffeine as an ergogenic aid prior to or during a sporting event, where endurance sports are the disciplines showing the highest urine caffeine excretion (and therefore prevalence) after competition [110, 115].

A recent meta-analysis reporting on 56 endurance time trials in athletes (79% cycling), found the percent difference between the caffeine and placebo group ranged from − 3.0 to 15.9% [195]. This wide range in performance outcomes highlights the substantial inter-individual variability in the magnitude of caffeine’s effects as reported. These inter-individual differences might be due to the methodological differences between the studies, habitual caffeine intake of the participants, and/or partly due to variation in genes that are associated with caffeine metabolism and caffeine response [213].

A recent systematic review was carried out on randomised placebo-controlled studies investigating the effects of caffeine on endurance performance and a meta-analysis was conducted to determine the ergogenic effect of caffeine on endurance time-trial performance [205]. Forty-six studies met the inclusion criteria and were included in the meta-analysis. This meta-analysis found that caffeine has a small but significant effect on endurance performance when taken in moderate doses (3–6 mg/kg) as well as an overall improvement following caffeine compared to placebo in mean power output of 2.9 ± 2.2% and a small effect size of 0.22 ± 0.15. Time-trial completion time showed improvements of 2.3 ± 2.6% with a small effect size of 0.28 ± 0.12. However, there was some variability in outcomes with responses to caffeine ingestion, with two studies reporting slower time-trial performance, and five studies reporting lower mean power output during the time–trial [205].

In summary, caffeine has been consistently shown to be effective as an ergogenic aid when taken in moderate doses (3–6 mg/kg), during endurance-type exercise and sport. Dozens of endurance studies are highlighted through this review is various sections, showing consistent yet wide-ranging magnitudes of benefit for endurance performance under caffeine conditions.

## Caffeine and muscular endurance, strength and power

Strength and power development through resistance exercise is a significant component of conditioning programs for both fitness and competitive sport. The most frequently consumed dose of caffeine in studies using strength tasks with trained or untrained individuals usually ranges from 3 to 6 mg/kg body mass (with 2 mg to 11 mg representing the entire range), ingested in the form of pills or capsules 30 to 90 min before exercise. In resistance exercise, strength is most commonly assessed using 1 repetition maximum (1RM) [214], or different isometric and isokinetic strength tests [215]. Muscular endurance assesses the muscle’s ability to resist fatigue and is an important quality in many athletic endeavors (e.g., swimming, rowing). Muscular endurance may be tested with repetitions of squats, maximal push-ups, bench press exercises (load corresponding to 60–70% of 1RM) to momentary muscular failure, or by isometric exercises such as the plank or static squat [216, 217].

Although several studies exploring the effects of caffeine on strength performance have been published since the 2010 ISSN caffeine position stand [40], some uncertainty surrounding the benefits of caffeine in activities involving muscular endurance, strength and power remains.

Caffeine was shown to be ergogenic for muscular endurance in two meta-analyses reporting effect sizes ranging from 0.28 to 0.38 (percent change range: 6 to 7%) [158, 218]. However, others have shown that it enhances strength but not muscular endurance [219, 220], and when studies have examined multiple strength-muscular endurance tasks, there were benefits across the board [67, 221], none at all [98, 222], or even impairments in muscular endurance with caffeine use [222, 223]. Ingesting caffeine prior to a muscular endurance task is likely to delay muscular fatigue, but these effects are not consistent among all studies.

Three meta-analyses explored the acute effects of caffeine on strength, and all reported ergogenic effects [158, 224, 225]. However, the effects in these meta-analyses were small, ranging from 0.16 to 0.20 (percent change: 2 to 7%). Such small improvements in muscular strength likely have the greatest practical meaningfulness for athletes competing in strength-based sports, such as powerlifting and weightlifting (athletes which already seem to be among the highest users of caffeine [110]).

Power output is often measured during a single-bout sprinting task using the Wingate test, which generally consists of ‘all-out’ cycling for 30 s performed at specific external loads (e.g., 7.5% of body mass). Power output is also assessed during different protocols of intermittent-sprinting and repeated-sprints often with the Wingate cycling test as well as assessments during running [226] or swimming repeated sprints [212].

The data for repeated sprint and power performance using Wingate data has been mixed. In an older study, 10 male team-sport athletes performed 18, 4-s sprints with 2-min active recovery [227]. Here, caffeine ingestion (6 mg/kg) enhanced mean power output and sprint work by 7 and 8.5%, respectively [227]. A more recent study examining the effects of acute caffeine ingestion on upper and lower body Wingate performance in 22 males did not report significant findings when measuring lower body mean and peak power using the Wingate test [228]. An older study by Greer et al. [229] also failed to report caffeine benefits on power output during a 30-s high-intensity cycling bout using the Wingate test. One meta-analysis reported that caffeine ingestion enhances mean and peak power during the Wingate test [230], although the effect sizes of 0.18 (+ 3%) and 0.27 (+ 4%), respectively are modest. In contrast, another meta-analysis that examined the effects of caffeine on muscle power as assessed with the Wingate test for three of the studies, and repeated sprints for a maximum of 10-s for the fourth, did not report benefits from ingestion of caffeine [231]. An average caffeine dose of 6.5 mg/kg of body mass was used across the four studies with no improvements in muscle power under caffeine conditions (effect size = 0.17, *p* = 0.36) compared to placebo trials, although the data collected spanned only 5 years [231]. A study by Lee et al. [232] reported that caffeine ingestion enhanced sprint performance involving a 90-s rest interval (i.e., intermittent-sprinting) but did not benefit repeated-sprints with a 20-s rest interval. This might suggest that the rest interval between sprints may modulate the ergogenic effects of caffeine. Indeed, a recent meta-analysis that focused on the effects of caffeine on repeated-sprint performance reported that total work, best sprint, and last sprint performance was not affected by caffeine ingestion [226].

Several studies have also shown substantial variability in outcomes. For example, one study [63] found that only 13 of 20 cyclists improved their performance with ~ 3–4 mg/kg of caffeine, while the remaining participants either worsened or did not alter their performance. Similarly, Woolf et al. [233] found that 5 mg/kg of caffeine improved overall peak power performance on the Wingate Test in 18 elite or professional athletes. However, 4 (28%) of the participants did not improve their performance with caffeine. Average power, minimum power, and power drop were not significantly different between treatments, but 72% of the participants obtained a greater peak power during the caffeine trial than during the placebo trial. There was also no overall improvement in average power or fatigue index, despite 13 (72%), and 9 (50%) of the participants, respectively, improving their performance. In summary, caffeine ingestion may be beneficial to enhance single and intermittent-sprint performance, while caffeine’s effects on repeated-sprint performance are inconsistent and require further research to draw stronger conclusions on the topic.

Ballistic movements (such as throws and jumps) are characterized by high motor unit firing rates, brief contraction times, and high rates of force development [234]. Many studies have explored the effects of caffeine on jumping performance [225, 235]. The body of evidence has indicated that caffeine supplementation increases vertical jump height during single and repeated jumps; however, the magnitude of these effects is rather modest, with effect sizes ranging from 0.17 to 0.22 (2 to 4%) [225, 235]. Besides jumping, several studies have explored the effects of caffeine on throwing performance. These studies reported that: (a) caffeine ingestion enhanced maximal shot put throwing distance in a group of 9 nine inter-collegiate track and field athletes [65]; and (b) caffeine ingestion at a dose of 6 mg/kg of body mass administered 60 min pre-exercise increased maximal medicine ball throwing distance [236]. Overall, the current body of evidence indicates that caffeine supplementation may be useful for acute improvements in ballistic exercise performance in the form of jumps and throws. However, more research is needed to explore the effects of caffeine on different throwing exercise tests, as this has been investigated only in a few studies.

Generally, the primary sports-related goal of strength and power-oriented resistance training programs is to move the force-velocity curve to the right, indicating an ability of the athlete to lift greater loads at higher velocities [237]. Several studies have explored the effects of caffeine on movement velocity and power in resistance exercise using measurement tools such as linear position transducers [238]. These studies generally report that caffeine ingestion provides ergogenic effects of moderate to large magnitudes, with similar effects noted for both mean and peak velocity, and in upper and lower-body exercises [67, 221, 239]. Even though this area merits further research to fill gaps in the literature, the initial evidence supports caffeine as an effective ergogenic aid for enhancing velocity and power in resistance exercise.

## Caffeine and sport-specific performance

Even though caffeine ingestion may enhance performance in the laboratory, there has been a paucity of evidence to support that these improvements transfer directly to sport-specific performance. To address this issue, several studies have also explored the effects of caffeine on sport-specific exercise tasks using sport simulation matches. Many studies conducted among athletes competing in team and individual sports, report that caffeine may enhance performance in a variety of sport tasks. However, there are also several studies that report no effects as outlined below:

- Basketball – increased jump height, but only in those with the AA version of the CYP1A2 gene [240], increased number of free throws attempted and free throws made, increased number of total and offensive rebounds [241], but did not improve sprint time [240], nor dribbling speed [242]
- Soccer – increased total distance covered during the game, increased passing accuracy, and jumping height [94, 243, 244], but the consumption of a caffeinated energy drink did not enhance performance in the “T test” in female soccer players [245], nor during match play in young football players [246]
- Volleyball – increased number of successful volleyball actions and decreased the number of imprecise actions [247, 248], although caffeine did not improve physical performance in multiple sport-specific tests in professional females [249], nor performance in volleyball competition [250]
- Football - did not improve performance for anaerobic exercise tests used at the NFL Combine [251]
- Rugby – increased the number of body impacts, running pace, and muscle power during jumping [252, 253], but did not impact agility [254]
- Field hockey – increased high-intensity running and sprinting [255], and may offset decrements in skilled performance associated with fatigue [256]
- Ice-hockey - has limited impact on sport-specific skill performance and RPE, but may enhance physicality during scrimmage [257]
- Combat sports – increased number of offensive actions and increased the number of throws [258]
- Cross-country skiing – reduced time to complete a set distance [259] and improved time to task failure [211]

In summary, although reviews of the literature show that caffeine ingestion is, *on average*, ergogenic for a wide range of sport-specific tasks, its use might not be appropriate for every athlete. Specifically, the use of caffeine needs to be balanced with the associated side-effects and therefore experimentation is required in order to determine the individual response before assessing whether the benefits outweigh the costs for the athlete. Athletes should gauge their physical response to caffeine during sport practice and competition in addition to monitoring mood state and potentially disrupted sleep patterns.

## Interindividual variation in response to caffeine

There is a lack of research examining potential interindividual differences in strength or anaerobic power-type exercise, but this is not the case for endurance exercise. In the myriad of studies examining caffeine on endurance performance, the benefits of caffeine do not appear to be influenced by sex, age, VO_2_ max, type of sport, or the (equivalent) dose of caffeine [13, 195, 260]. Nevertheless, there appears to be substantial interindividual variability in response to caffeine under exercise conditions, which may be attributed to several factors outlined below.

### Genetics

Genetic variants affect the way we absorb, metabolize, and utilize and excrete nutrients, and gene-diet interactions that affect metabolic pathways relevant to health and performance are now widely recognized [261]. In the field of nutrigenomics, caffeine is the most widely researched compound with several randomized controlled trials investigating the modifying effects of genetic variation on exercise performance [75, 208, 262, 263].

Numerous studies have investigated the effect of supplemental caffeine on exercise performance, but there is considerable inter-individual variability in the magnitude of these effects [11, 13, 44] or in the lack of an effect [264, 265], when compared to placebo. Due to infrequent reporting of individual data it is difficult to determine the extent to which variation in responses may be occurring. The performance of some individuals is often in stark contrast to the average findings reported, which may conclude beneficial, detrimental, or no effect of caffeine on performance. For example, Roelands et al. [265] reported no ergogenic effect of caffeine in a study involving trained male cyclists. The authors concluded that inter-individual differences in response to caffeine might be responsible for the lack of overall performance improvement, as 50% of subjects improved while 50% worsened, in the caffeine compared to the placebo trial.

These inter-individual differences appear to be partly due to variations in genes such as *CYP1A2* and possibly *ADORA2A*, which are associated with caffeine metabolism, sensitivity and response [213]. Over 95% of caffeine is metabolized by the CYP1A2 enzyme, which is encoded by the *CYP1A2* gene and is involved in the demethylation of caffeine into the primary metabolites paraxanthine, theophylline and theobromine [127]. The -163A > C (rs762551) single nucleotide polymorphism (SNP) has been shown to alter CYP1A2 enzyme inducibility and activity [132, 134], and has been used to categorize individuals as ‘fast’ or ‘slow’ metabolizers of caffeine. In the general population, individuals with the AC or CC genotype (slow metabolizers) have an elevated risk of myocardial infarction [266], hypertension and elevated blood pressure [267, 268], and pre-diabetes [269], with increasing caffeinated coffee consumption, whereas those with the AA genotype show no such risk. Additionally, regular physical activity appears to attenuate the increase in blood pressure induced by caffeine ingestion, but only in individuals with the AA genotype [268].

The largest caffeine, genetics and exercise study to date [208] examined the effects of caffeine and *CYP1A2* genotype on 10-km cycling time trial performance in competitive male athletes (both endurance and power sports) after ingestion of placebo, and caffeine doses of 2 mg (low dose) or 4 mg (moderate dose) per kg body mass. There was a 3% improvement in cycling time with the moderate dose in all subjects, which is consistent with previous studies using similar doses [13, 206]. However, there was a significant caffeine-gene interaction; improvements in performance were seen at both caffeine doses, but only in those with the AA genotype who are ‘fast metabolizers’ of caffeine. In that group, a 6.8% improvement in cycling time was observed at 4 mg/kg, which is greater than the 2–4% mean improvement seen in several other studies using cycling time trials and similar doses [13, 201, 206, 207, 270–272]. Among those with the CC genotype (i.e., “slow metabolizers”), 4 mg/kg caffeine impaired performance by 13.7%, whereas no difference was observed between the placebo and 2 mg/kg caffeine trials. In those with the AC genotype there was no effect of either dose [208]. The findings are consistent with a previous study [263] that observed a caffeine-gene interaction indicating improved time trial cycling performance following caffeine consumption only in those with the AA genotype.

In contrast, previous studies either did not observe any impact of the *CYP1A2* gene in caffeine-exercise studies [273, 274], or reported benefits only in slow metabolizers [75]. There are several reasons that may explain discrepancies in study outcomes. These include smaller samples sizes with few and/or no subjects in one genotype [75, 273, 274], as well as shorter distances or different types of performance test (power versus endurance) [75] compared to the aforementioned trials, which reported improved endurance after caffeine ingestion in those with the *CYP1A2* AA genotype [208, 263]. The effects of genotype on performance might be the most prominent during training or competition of longer duration or an accumulation of fatigue (aerobic or muscular endurance) [149], where caffeine appears to provide its greatest benefits, and where the adverse effects to slow metabolizers are more likely to manifest [195, 260]. Indeed, in a study of performance in elite basketball players [240], only in those with the AA genotype caffeine improved repeated jumps which requires maintaining velocity at take-off repeatedly as an athlete fatigues throughout a game (muscular endurance) - even though there was no caffeine-genotype interaction effect for this outcome. However, caffeine similarly improved performance in those with the both AA and C-genotypes during a simulated basketball game [240]. In a cross-over design of 30 resistance-trained men, caffeine ingestion resulted in a higher number of repetitions in repeated sets of three different exercises, and for total repetitions in all resistance exercises combined, which resulted in a greater volume of work compared to placebo conditions, but only in those with the *CYP1A2* AA genotype [262]. Although more research is warranted, there is a growing body of evidence to support the role of *CYP1A2* in modifying the effects of caffeine ingestion on aerobic or muscular endurance-type exercise, which helps to determine which athletes are most likely to benefit from caffeine.

*The ADORA2A* gene is another genetic modifier of the effects of caffeine on performance. The adenosine A_2A_ receptor, encoded by the *ADORA2A* gene, has been shown to regulate myocardial oxygen demand and increase coronary circulation by vasodilation [275, 276]. The A_2A_ receptor is also expressed in the brain, where it has significant roles in the regulation of glutamate and dopamine release, with associated effects on insomnia and pain [277, 278]. The antagonism of adenosine receptors after caffeine ingestion is modified by the *ADORA2A* gene, which may allow greater improvements in dopamine transmission and lead to norepinephrine and epinephrine release due to increased neuronal firing [168] in some genotypes versus others. Dopamine has been associated with motivation and effort in exercising individuals, and this may be the mechanism by which differences in response to caffeine are manifested [141, 168, 169].

Currently, only one small pilot study has examined the effect of the *ADORA2A gene* (rs5751876) on the ergogenic effects of caffeine under exercise conditions [279]. Twelve female subjects underwent a double-blinded, crossover trial comprising two 10-min cycling time trials following caffeine ingestion or placebo. Caffeine benefitted all six subjects with the TT genotype, but only one of the six C allele carriers. Further studies are needed to confirm these preliminary findings and should include a large enough sample to distinguish any effects between the different C allele carriers (i.e. CT vs. CC genotypes) and potential effects related to sex.

The *ADORA2A* rs5751876 genotype has also been implicated, by both objective and subjective measures, in various parameters of sleep quality after caffeine ingestion in several studies [280–283]. Adenosine promotes sleep by binding to its receptors in the brain, mainly A_1_ and A_2A_ receptors, and caffeine exerts an antagonist effect, blocking the receptor and reversing the effects of adenosine and promoting wakefulness [280]. This action, as well as the potency of caffeine to restore performance (cognitive or physical) in ecological situations, such as highway-driving during the night [284], supports the notion that the adenosine neuromodulator/receptor system is significantly involved in sleep–wake regulation. This action of caffeine may also serve athletes well under conditions of jetlag, and irregular or early training or competition schedules. Psychomotor speed relies on the ability to respond, rapidly and reliably, to randomly occurring stimuli which is a critical component of, and characteristic of, most sports [285]. Genetic variation in *ADORA2A* has been shown to be a relevant determinant of psychomotor vigilance in the rested and sleep-deprived state and modulates individual responses to caffeine after sleep deprivation [282]. Those with the CC genotype of *ADORA2A* rs5751876 consistently performed on a higher level on the sustained vigilant attention task than T-allele -carriers; however, this was tested in *ADORA2A* haplotypes that included combinations of 8 SNPs. This work provides the basis for future genetic studies of sleep using individual *ADORA2A* SNPs.

As mentioned, the *ADORA2A* genotype has also been implicated in sleep quality and increases in sleep disturbance [283]. Consistent with the “adenosine hypothesis” of sleep where the accumulation of adenosine in the brain increases sleep propensity, caffeine prolongs time to fall asleep, decreases the deep stages of non-rapid-eye movement (nonREM) sleep, reduces sleep efficiency, and alters the waking and sleep electroencephalogram (EEG) frequencies, which reliably reflect the need for sleep [286–288]. Increased beta activity in nonREM sleep may characterize individuals with insomnia when compared with healthy good sleepers [289]. A functional relationship between the *ADORA2A* genotype and the effect of caffeine on EEG beta activity in nonREM sleep has previously been reported [281], where the highest rise was in individuals with the CC genotype, approximately half in the CT genotype, whereas no change was present in the TT genotype. Consistent with this observation, the same study found individuals with the CC and TC genotypes appeared to confer greater sensitivity towards caffeine-induced sleep disturbance compared to the TT genotype [281]. This suggests that a common variant in *ADORA2A* contributes to subjective and objective responses to caffeine on sleep.

### Caffeine, genetics and anxiety

In elite athletes, 50% face mental health issues sometime during their career [290]. Given that anxiety may be normalized in elite sports even at clinical levels, factors that contribute to anxiety should be mitigated whenever possible. Anxiety may be caused by stress-related disorders (burnout), poor quality sleep patterns (often related to caffeine intakes) and possibly as a response to caffeine ingestion due to genetic variation, even at low levels [109].

As previously mentioned, caffeine blocks adenosine receptors, resulting in the stimulating effects of caffeine [213]. A common variation in the *ADORA2A* (adenosine A_2A_ receptor) gene contributes to the differences in subjective feelings of anxiety after caffeine ingestion [291, 292], especially in those who are habitually low caffeine consumers [293]. This may be particularly relevant to athletes who possess the TT variant of rs5751876 in the *ADORA2A* gene. These individuals are likely to be more sensitive to the stimulating effects of caffeine and experience greater increases in feelings of anxiety after caffeine intake than do individuals with either the CT or CC variant [291–293].

Sport psychologists commonly work with athletes to help them overcome anxiety about performance during competitions. Anxiety before or during athletic competitions can interfere not only in performance, but also in increased injury risk [294]. Athletes who are more prone to performance anxiety may exacerbate their risk for feelings of anxiety depending on their caffeine use and which variant of the *ADORA2A* gene they possess. Monitoring the actions of caffeine in those individuals who are susceptible, may alleviate some of the related feelings of anxiety with caffeine use. Given that anxiety may disrupt concentration and sleep and negatively impact social interactions, athletes with higher risks and prevalence for anxiety, may want to limit or avoid caffeine consumption (if caffeine is a known trigger) during times where they are feeling anxious or stressed, such as at sporting competitions or social gatherings or other work and school events.

The importance of both sleep and caffeine (as an ergogenic aid) to athletes highlights the importance of optimizing rest and recovery through a better understanding of which athletes may be at greater risk of adverse effects of caffeine on mood and sleep quality, possibly due to genetic variation. This information will allow athletes and coaching staff to make informed decisions on when and if to use caffeine when proximity to sleep is a factor. These considerations will also be in conjunction with the possibility that an athlete will benefit from caffeine in endurance-based exercise as determined in part, by their *CYP1A2* genotype, albeit with a clear need for future research.

### Habitual caffeine intake

The quantification of habitual caffeine intake is difficult, which is problematic for studies aiming to compare performance outcomes following caffeine ingestion in habitual versus non-habitual caffeine users. This concern is highlighted by reports showing large variability in the caffeine content of commonly consumed beverages, e.g. ~ 8- to 9-fold differences in caffeine content have been reported in coffee beverages purchased from similar retail shops [295] and in pre-workout supplements [296]. Self-reported intakes may therefore be unreliable. Newly discovered biomarkers of coffee consumption may be more useful for quantifying intakes in the future, but currently, these are not widely available [297]. Different protocols for the length of the caffeine abstinence period preceding data collection is also a relevant factor in determining variability in performance outcomes. For example, in shorter caffeine abstinence periods e.g., 12–48 h, reversal of caffeine withdrawal effects by acute caffeine supplementation may have positive effects on performance, i.e. alleviating the negative symptoms of withdrawal, which in itself may improve performance [298]. These effects may be more pronounced in those genetically predisposed to severe withdrawal effects [299]. However, in one study 3 mg/kg caffeine significantly improved exercise performance in trained cyclists (*n* = 12), irrespective of whether a 4-day withdrawal period was imposed on habitual caffeine users [300]. Another study also reported increased endurance in habitual caffeine users (*n* = 6) regardless of a 0, 2- or 4-day abstinence period. The authors concluded that improved performance under caffeine conditions at 6 mg/kg is not related to prior caffeine habituation in recreational athletes [301]. Although genes have been associated with habitual caffeine intake using GWAS research [302, 303], it is important to highlight that these associations are not directly applicable to determining differences in performance outcomes in response to acute caffeine doses for regular or habitual caffeine users versus non-habitual users. The “caffeine habits” of individuals are more likely related to their personal experience with adverse effects such as feel jittery, experiencing tachycardia or insomnia. Furthermore, associations between genes and habitual caffeine intake do not elucidate potential mechanisms by which caffeine intake behaviors may influence subsequent performance following caffeine supplementation [304, 305]. In animal model studies, regular consumption of caffeine has been associated with an upregulation of the number of adenosine receptors in the vascular and neural tissues of the brain [306]. Although, this did not appear to modify the effects of caffeine in one study [307], in another, chronic caffeine ingestion by mice caused a marked reduction in locomotor exploratory activity [308]. Changes in adenosine receptor number or activity have not been studied in humans.

There does not appear to be a consistent difference in the performance effects of acute caffeine ingestion between habitual and non-habitual caffeine users, and study findings remain equivocal. In one study, habitual stimulation from caffeine resulted in a general dampening of the epinephrine response to both caffeine and exercise; however, there was no evidence that this impacted exercise performance [309]. Another study [310] examined the effect of 4 weeks of caffeine supplementation on endurance performance in 18 low-habitual caffeine consumers who were randomly assigned to ingest caffeine or placebo for 28 days. Four weeks of caffeine ingestion resulted in increased tolerance to acute caffeine supplementation in previously low habitual caffeine consumers, with the ergogenic effect of acute caffeine supplementation no longer apparent [310]. These results are in contrast with a recent study in which 20 days of consecutive supplementation with caffeine maintained an ergogenic effect, even though the effect size attenuated over time [311]. More recently, a double-blind, crossover, counterbalanced study was performed [312], where 40 endurance-trained male cyclists were allocated into tertiles according to their daily caffeine intake: low (58 ± 29 mg), moderate (143 ± 25 mg), and high consumers (351 ± 139 mg). Participants completed three trials in which they performed simulated cycling time-trials under three conditions: caffeine (6 mg/kg), placebo, and no supplement (control). Caffeine ingestion improved performance as compared to placebo and control, with no influence of habitual caffeine intake. Additionally, no correlation was observed between habitual caffeine intake and absolute changes in a ~ 30 min cycling time-trial performance with caffeine [312]. However, a limitation of this study is the short 24-h caffeine withdrawal period in all groups which may have resulted in performance improvements due to the reversal of caffeine withdrawal effects, rather than impact of acute-on-chronic caffeine administration and the effects of habituation to caffeine on exercise performance [298, 313]. In addition, habitual caffeine intake was estimated using a food frequency questionnaire, which might be a limitation given the already mentioned variation of caffeine in coffee and different supplements.

There is wide variability in caffeine content of commonly consumed items, and as such, an objective measure (e.g., caffeine or metabolite levels) might be considered to reported caffeine intakes [297, 313]. Based on these observations, the assumption that habitual and nonhabitual caffeine consumers will or will not respond differently to caffeine supplementation during exercise, requires further study.

### Caffeine timing

The most common timing of caffeine supplementation is 60 min before exercise. This timing is used given that it is believed that 60 min post-ingestion, plasma levels of caffeine are at maximal values [314]. However, caffeine appears to be most beneficial during times or in sports where there is an accumulation of fatigue, i.e., exercise over a longer continuous or intermittent duration [64]. Therefore, ingestion of caffeine during exercise (mid/later stages) may be more beneficial than ingestion beforehand for some individuals depending upon the length of the event. A recent review [195] reported that the effect size of caffeine benefits increase with the increasing duration of the time trial event, meaning that timing caffeine intake closer to a time of greater fatigue, i.e., later in the race, may be most beneficial. This supports the notion that endurance athletes (with longer races) may benefit most from caffeine for performance enhancement since they have the greatest likelihood of being fatigued. This also supports findings in other investigations that show ingesting caffeine at various time points including late in exercise may be most beneficial [196].

For example, an early study [196] aimed to understand whether or not there were benefits to a common practice among endurance athletes, such as those participating in marathons and triathlons, which is to drink flat cola toward the end of an event. When researchers investigated the ingestion of a low dose of caffeine toward the end of a race (e.g., in the form of flat cola) it was found to have comparable effects as ingesting higher doses, such as ~ 5 or 6 mg/kg, ingested ~ 60 min before the race. The study also demonstrated that the effect was due to the caffeine and not the carbohydrate, which may also aid performance as fuel stores become depleted [196].

More recently, caffeine gum ingestion enhanced cycling performance when it was administered immediately prior to exercise, but not when administered 1 or 2 h beforehand. This may have been due to the faster absorption with caffeinated gum consumption, and due to the continued increase in plasma caffeine concentrations during the cycling time trial, when athletes may become fatigued (i.e. 30 + minutes into exercise), as the trials also included a 15 min steady-state cycling bout prior to the time trial [60]. Similarly, in a lab setting, a study of athletes completing 120 min of steady-state cycling followed by a time trial under conditions of placebo and caffeine, found that the ingestion of both low and moderate doses of caffeine later in exercise were beneficial [149]. However, there was significant interindividual variability, highlighting the need for athletes to experiment with their own strategies as far as dosing and timing are concerned.

The optimal timing of caffeine ingestion may depend on the source of caffeine. As stated earlier, some of the alternate sources of caffeine such as caffeine chewing gums may absorb more quickly than caffeine ingested in caffeine-containing capsules [60]. Therefore, individuals interested in supplementing with caffeine should consider that timing of caffeine ingestion will likely be influenced by the source of caffeine.

### Training status

Training status may mediate the magnitude of caffeine’s ergogenic effect, but studies have reported mixed results. Although a 2010 meta-analysis [158] did not find differences (*p* = 0.08) in caffeine’s ability to enhance muscle endurance in untrained subjects versus trained subjects, these results were not derived from direct comparisons between trained and untrained subjects. Currently, only a few investigations [96, 210, 315–318] have included both trained and untrained subjects in their study design.

In a study of elite and occasional swimmers [318], it was reported that 250 mg of supplemental caffeine was ergogenic only for competitive swimmers and not recreational swimmers. A limitation of this study is that the swimming exercise task differed between the trained and untrained participants. Specifically, the study utilized 1600-m swimming for the trained swimmers and 400-m for the untrained swimmers, which is a likely explanation for these findings. However, some have also postulated that this is because athletes perform more reliably on a given task than nonathletes, and increased test-retest reliability might prevent type II errors [319]. In contrast to the above evidence regarding the importance of training status, other research has shown that training status does not moderate the ergogenic effects of caffeine on exercise performance. One study [210] showed similar performance improvements (1.0 and 1.1%) in 15 well-trained and 15 recreational runners performing an outdoor 5 km time trial after 5 mg/kg caffeine intake compared to the placebo trial. Similarly, Astorino et al. [96] found that overall, acute caffeine intake improved 10 km time-trial performance in both endurance-trained athletes and active men, with no differences seen between groups. Likewise, an investigation concluded that there was no ergogenic effect of caffeine at a dose of 5 mg/kg on time to exhaustion in either endurance trained or untrained men [315].

More recently, a small study by Boyett et al. [317] investigated the interactions of 6 mg/kg caffeine on training status and time of day in 20 male subjects. Subjects completed four experimental trials consisting of a 3-km cycling time trial performed in randomized order for each combination of time of day (morning and evening) and treatment. They reported that both untrained and trained subjects improved performance with caffeine supplementation in the morning; however, only the untrained subjects improved when tested in the evening. Although there were some limitations to this study, these observations indicate that trained athletes are more likely to experience ergogenic effects from caffeine in the morning, while untrained individuals appear to receive larger gains from caffeine in the evening than their trained counterparts. This may further complicate the training status data with a possible temporal effect [317]. The concentration of adenosine receptors (the primary target of caffeine) do appear to be higher in trained compared to untrained individuals, but this has only been reported in animal studies [320]. Boyett et al. [317] speculated that the higher concentration of adenosine receptors may increase tissue sensitivity to any given concentration of adenosine.

Although some studies comparing training status of subjects support the notion [318] that training influences response to caffeine during exercise, most do not [96, 210, 315] and this was also the finding in a subsequent meta-analysis [158]. It is possible that the only difference between trained and untrained individuals is that trained individuals likely have the mental discipline to exercise long or hard enough to benefit more from the caffeine stimulus, which might provide an explanation for why in some studies, trained individuals respond better to caffeine [314]. Currently, it seems that trained and untrained individuals experience similar improvements in performance following caffeine ingestion; however, more research in this area is warranted.

### Caffeine and sleep

The impacts of caffeine on sleep and behavior after sleep deprivation are widely reported [321]. Sleep is recognized as an essential component of physiological and psychological recovery from, and preparation for, high-intensity training in athletes [322, 323]. Chronic mild to moderate sleep deprivation in athletes, potentially attributed to caffeine intakes, may result in negative or altered impacts on glucose metabolism, neuroendocrine function, appetite, food intake and protein synthesis, as well as attention, learning and memory [323]. These factors can all influence an athlete’s nutritional, metabolic, and endocrine status negatively and hence potentially affect energy levels, muscle repair, immunity, body composition, memory and learning and result in diminished athletic performance [324, 325].

Objective sleep measures using actigraphy or carried out in laboratory conditions with EEG have shown that caffeine negatively impacts several aspects of sleep quality such as: sleep latency (time to fall asleep), WASO (wake time after sleep onset), sleep efficiency and duration [321]. Studies in athletes have also shown adverse effects in sleep quality and markers for exercise recovery after a variety of doses of caffeine ingestion [326–328]. Although caffeine is associated with sleep disturbances, caffeine has also been shown to improve vigilance and reaction time and improved physical performance after sleep deprivation [282, 329–332]. This may be beneficial for athletes or those in the military who are traveling or involved in multiday operations, or sporting events and must perform at the highest level under sleep-deprived conditions [192, 194, 330, 332].

Even though caffeine ingestion may hinder sleep quality, the time of day at which caffeine is ingested will likely determine the incidence of these negative effects. For example, in one study that included a sample size of 13 participants, ingestion of caffeine in the morning hours negatively affected sleep only in one participant [333]. However, ingestion of caffeine in the late afternoon (18:00 h) resulted in insomnia effects among 6 participants. These results are likely explained by the half-life of caffeine, which is generally around 4 to 6 h (even though it varies between individuals). Unfortunately, athletes and those in the military are unlikely to be able to make adjustments to the timing of training, competition and military exercises or the ability to be combat ready. However, to help avoid negative effects on sleep, athletes may consider using caffeine earlier in the day whenever possible. Pronounced individual differences have also been reported where functional genetic polymorphisms have been implicated in contributing to individual sensitivity to sleep disruption [280, 281] and caffeine impacts after sleep deprivation [282] as discussed in the *Interindividual variation in response to caffeine: Genetics* section of this paper.

### Side-effects associated with caffeine intake

As with any supplement, caffeine ingestion is also associated with certain side-effects. Some of the most commonly reported side-effects in the literature are tachycardia and heart palpitations, anxiety [281, 291], headaches, as well as insomnia and hindered sleep quality [239, 326]. For example, in one study, caffeine ingestion before an evening Super Rugby game resulted in a delay in time at sleep onset and a reduction in sleep duration on the night of the game [327]. Caffeine ingestion is also associated with increased anxiety; therefore, its ingestion before competitions in athletes may exacerbate feelings of anxiety and negatively impact overall performance (see caffeine and anxiety section). Increased jitters/anxiety/arousal associated with caffeine ingestion also needs to be considered within the specific demands of each sport, and even the position within a given sport. For example, athletes competing in sports that heavily rely on the skill component (e.g., tennis players, biathlon shooting) would likely not benefit from caffeine-induced jitters and arousal. However, athletes in sports that depend more on physical capabilities, such as strength and endurance (e.g., football lineman), might actually benefit from increased jitters and arousal before games. These aspects are less explored in research but certainly warrant consideration in the practical context to optimize the response to caffeine supplementation. The primary determinant in the incidence and severity of side-effects associated with caffeine ingestion is the dose used. Side-effects with caffeine seem to increase linearly with the dose ingested [239]. Therefore, they can be minimized—but likely not fully eliminated—by using smaller doses, as such doses are also found to be ergogenic and produce substantially fewer side-effects [112]. In summary, an individual case-by-case basis approach is warranted when it comes to caffeine supplementation, as its potential to enhance performance (benefit) needs to be balanced with the side-effects (risk).

### Caffeine and cognitive performance

In addition to exercise performance, caffeine has also been studied for its contribution to athletes of all types (including Special Forces operators in the military) who are routinely required to undergo periods of sustained cognitive function and vigilance due to their job requirements (Table 1). A 2016 review [344] concluded that caffeine in doses from 32 to 300 mg (for a 75 kg individual) enhanced specific aspects of cognitive performance, such as attention, vigilance, and reaction time. Spriet [112] also concluded that lower doses of caffeine (approximately 200 mg) improved cognitive processes associated with exercise including vigilance, alertness, and mood. Hogervorst et al. [82] studied 24 well-trained cyclists that were randomized to 3 groups: (1) consumed a bar containing 45 g of carbohydrate and 100 mg of caffeine; (2) an isocaloric non-caffeine performance bar; or, (3) a placebo beverage (non-caloric flavored water) immediately before performing a 2.5-h ride followed by a time to exhaustion trial. They found that caffeine in a carbohydrate-containing performance bar significantly improved both endurance performance and complex cognitive ability during and after exercise [82]. Antonio et al. [345] assessed the effects of an energy drink on psychomotor vigilance in a small cohort of 20 exercise-trained men and women. The acute consumption of 300 mg of caffeine in a commercially available energy drink produced a significant improvement in psychomotor vigilance mean reaction time in these subjects compared to the placebo trial. This matches a 2001 IOM report [346] that the effects of caffeine supplementation include increased attention and vigilance, complex reaction time, and problem-solving and reasoning.

**Table 1:** Summary of studies that explored the effects of caffeine on cognitive function

Author	Participants	Protocol	Outcome
**Sleep Deprived**			
Hogervorst et al. 2008 [82]	Well-trained cyclists (*n* = 24)	• **Bar with 100 mg caffeine and 45.0 g CHO** • Bar with only 45.0 g CHO • 300 mL non-caloric beverage	*↑Stroop and Rapid Visual Information Processing tests after 140 min and time to exhaustion exercise trial at 75% VO2_max_
McLellan et al. 2007 [334]	Soldiers (*n* = 20)	• **600 mg total caffeine in 200 mg does over 6 h period** • Placebo	*↑ Increased vigilance
McLellan et al. 2005 [329]	Soldiers (*n* = 31)	• **200 mg caffeine (gum) mg doses over 5 h** • Placebo	Maintained vigilance in control observation and reconnaissance vigilance task
McLellan et al. 2005 [330]	Soldiers (*n* = 30)	• **600 mg total caffeine in 100 mg and 200 mg doses over a 6 h period** • Placebo	Sustained marksmanship vigilance and accuracy *less decrease in urban operations vigilance
Lieberman et al. 2002 [42]	U.S. Navy SEAL trainees (*n* = 68)	• 100 mg caffeine • **200 mg caffeine** • **300 mg caffeine** • Placebo	*↑improved vigilance and reaction time in both the 200 and 300 mg caffeine interventions following 72 h sleep deprivation
Kamimori et al. 2015 [332]	Special Forces Operators (*n* = 20)	• **Four 200 mg doses of caffeine** • Placebo	*maintained psychomotor speed, improved event detection, increased the number of correct responses to stimuli, and increased response speed during logical reasoning tests. ⬌Live-fire marksmanship was not altered by caffeine.
Tikuisis et al. 2004 [335]	Young Military Subjects (*n* = 20)	• 400 mg caffeine • 100 mg caffeine • 100 mg of caffeine • Placebo	*increased cognitive component of shooting task
**Not Sleep Deprived**			
Share et al. 2009 [336]	Elite male shooters (*n* = 7)	• 2 mg/kg caffeine • 4 mg/kg caffeine • Placebo	⬌ shooting accuracy, reaction time, or target tracking time between groups
Pomportes et al. 2019 [337]	Modern pentathlon national team athletes (*n* = 10)	• Four counterbalanced sessions with: • 30 g CHO • **300 mg guarana complex** • **200 mg caffeine** • Placebo	* enhanced speed of information processing w CHO, and caffeine and guarana complex * lower RPE w caffeine and gaurana complex
Duncan et al. 2019 [228, 338]	Younger males (*n* = 12)	• **5 mg/kg dose caffeine** • Placebo 60 min before 30 s upper body Wingate anaerobic test	*Readiness to invest physical effort, and cognitive performance *Reduced rating of perceived exertion ⬌Response accuracy
**Other Stressors**			
Share et al. 2009 [336]	Elite male shooters (*n* = 7)	• 2 mg/kg caffeine • 4 mg/kg caffeine • Placebo	⬌ shoot accuracy, reaction time, or target tracking time between groups
Gillingham et al. 2004 [339]	Military reservists (*n* = 12)	• **5 mg/kg caffeine** or placebo dosed before 2.5 h loaded march plus 1 h sandbag wall construction task then **re-dose of 2.5 mg/kg caffeine** or placebo	*↑ marksmanship performance (engagement time and number of shots fired) ⬌friend-foe discrimination
Zhang et al. 2014 [340]	Firefighters (*n* = 10)	• 400 mg caffeine • Menthol lozenges • Placebo	⬌ Change in perceived exertion, mood reaction time, short-term memory, or retrieval memory
Crowe et al. 2006 [341]	Healthy subjects: male (*n* = 12) female (*n* = 5)	• 6 mg/kg caffeine • Placebo	⬌ rating of perceived exertion
Foskett et al. 2009 [244]	Male soccer players (*n* = 12)	• **6 mg/kg of caffeine** • Placebo	* Enhanced fine motor skills via improved ball passing accuracy and control
Stuart et al. 2005 [342]	Competitive male rugby (*n* = 9)	• **6 mg/kg caffeine** • Placebo	*Increased ball-passing accuracy
Duvnjak-Zaknich et al. 2011 [343]	Moderately trained male athletes (*n* = 10)	• 6 mg/kg caffeine • Placebo	*Main effect for condition on decision time

Outcomes are bold caffeine group specific; * = significant difference, ↑ = improved performance, ⬌ no change, mg/kg = milligram per kilogram, CHO = carbohydrate

One confounding factor on cognitive effects of caffeine is the role of sleep. Special Forces military athletes conduct operations where sleep deprivation is common. A series of different experiments [42, 329, 330, 332, 334, 335, 346, 347] have examined the effects of caffeine in real-life military conditions. In three of the studies [329, 330, 334], soldiers performed a series of tasks such as a 4 or 6.3 km run and a marksmanship test, which is a task that requires fine motor coordination and steadiness, observation/reconnaissance, and requires long periods of no movement coupled with alertness and psychomotor vigilance over several days, where opportunities for sleep became more infrequent. Caffeine was provided at doses ranging from 600 to 800 mg in the form of chewing gum, owing to its practicality, i.e., rapid absorption and portability [58]. The investigators found that vigilance was either maintained or enhanced under the caffeine conditions (vs. placebo), in addition to improvements in run times and obstacle course completion [329, 330, 334]. Similarly, Lieberman et al. [42] examined the effects of caffeine on cognitive performance during sleep deprivation in U. S. Navy Seals. During this investigation, there were multiple doses of caffeine ingested, 100 mg, 200 mg, or 300 mg, in capsule form. Once again, results were also significant for the assessments related to vigilance and reaction time in both the 200 and 300 mg caffeine intervention, suggesting smaller successive doses of caffeine are more beneficial than large boluses, for improving focus and vigilance.

The positive effects of caffeine on cognitive function were further supported by work from Kamimori et al. [332] where 20 special forces operators were randomly assigned to receive four 200-mg doses of caffeine or placebo during a period of low sleep over three successive days. The caffeine intervention maintained psychomotor speed, improved event detection, increased the number of correct responses to stimuli, and increased response speed during logical reasoning tests. Under similar conditions of sleep deprivation, Tikuisis et al. [335] demonstrated that the cognitive component of a shooting task (i.e., target detection) benefited from caffeine. These studies [42, 329, 330, 332, 334, 335, 346, 347] demonstrate the effects of caffeine on vigilance and reaction time in a sleep deprived state, in a distinct and highly trained population, usually with repeated ‘lower’ doses, ~ 200 mg of caffeine ingestion. When subjects are not sleep deprived, the effects of caffeine on cognition appear to be less effective. For example, Share et al. [336] did not show any difference in shooting accuracy, reaction time, or target tracking times among the three intervention trials using 2 escalating doses of caffeine at 2 mg/kg and 4 mg/kg.

In addition to the ability of caffeine to counteract the stress from sleep deprivation, it may also play a role in combatting other stressors. Gillingham et al. [339] showed that in 12 reservists who ingested 5 mg/kg body mass of caffeine or placebo 1 h pre- and post-strenuous exercise, that caffeine ingestion mediated stress from sleep deprivation cited above. However, these benefits were not observed during more complex operations [339]. With a different stressor (a simulated firefight), no cognitive effect was seen with a caffeine dose of 400 mg [340]. Crowe et al. [341] examined the effects of caffeine (6 mg/kg dose) on cognitive parameters (visual reaction time and number recall tests) via two maximal 60-s bouts of cycling over three conditions (caffeine, placebo, control). Again, no cognitive benefit was observed.

Other studies [244, 338, 342, 343] support the effects of caffeine on the cognitive aspects of sport performance, even though with some mixed results [348, 349]. Foskett et al. [244] determined that a moderate dose (6 mg/kg) of caffeine enhanced the fine motor skills in soccer players as measured by improved ball passing accuracy and control. This was supported by Stuart et al. [342] who examined the effects of the same dose of caffeine (6 mg/kg) and found a 10% improvement in ball-passing accuracy. Equivocal results were reported for distance covered, agility, and accuracy in a review of 19 studies where caffeine ingestion before exercise was between 3 and 6 mg/kg [348]. Data on reactive agility time is split, with one study demonstrating a benefit [343] and another one [349] did not showing any benefit, despite using the same dose of caffeine (6 mg/kg). Finally, caffeine (5 mg/kg) was shown to enhance cognitive performance after an upper-body Wingate test, which may be beneficial in sports or occupational activities where there is a need for anaerobic performance concurrent with decision making (e.g. firefighting, military related tasks, wheelchair basketball) [338].

The exact mechanism of how caffeine enhances cognition in relation to exercise is not fully elucidated and appears to work through both peripheral and central neural effects [350]. In a study by Lieberman et al. [42], 8 h after caffeine administration, caffeine continued to enhance motor learning and short-term memory via performance of repeated acquisition. Repeated acquisition are behavioral tests in which subjects are required to learn new response sequences within each experimental session [351]. The researchers [42] speculated that caffeine exerted its effects from an increased ability to sustain concentration, as opposed to an actual effect on working memory. Other data [352] were in agreement that caffeine reduced reaction times via an effect on perceptual-attentional processes (not motor processes). This is in direct contrast to earlier work that cited primarily a motor effect [353]. Another study with a sugar free energy drink showed similar improvements in reaction time in the caffeinated arm; however, they attributed it to parallel changes in cortical excitability at rest, prior, and after a non-fatiguing muscle contraction [354]. The exact cognitive mechanism(s) of caffeine have yet to be elucidated.

Based on some of the research cited above, it appears that caffeine is an effective ergogenic aid for individuals either involved in special force military units or who may routinely undergo stress including, but not limited to, extended periods of sleep deprivation. Caffeine in these conditions has been shown to enhance cognitive parameters of concentration and alertness. It has been shown that caffeine may also benefit sport performance via enhanced passing accuracy and agility. However, not all of the research is in agreement. It is unlikely that caffeine would be more effective than actually sleeping, i.e. you cannot ‘outcaffeinate’ poor sleep.

### Environmental influences on response to caffeine

Physical activity and exercise in extreme environments are of great interest as major sporting events (e.g. Tour de France, Leadville 100, Badwater Ultramarathon) are commonly held in extreme environmental conditions. Events that take place in the heat or at high altitudes bring additional physiological challenges (i.e., cardiovascular strain, thermoregulation, diuresis) for athletes, which may be potentially compounded if caffeine is consumed prior to and/or during training or competition in such environments [355]. Nonetheless, caffeine is widely used by athletes as an ergogenic aid when exercising or performing in extreme environmental situations. The current understanding of caffeine’s impact on exercise performance is based largely on the findings of analyses conducted in controlled, temperate environments, whereas data collected on the ergogenic effect of caffeine consumption by individuals performing in the heat or at altitude are limited and have resulted in inconsistent findings.

#### Heat

The ability to perform prolonged exercise is impaired in hot and/or humid environments [355, 356]. The use of caffeine in conjunction with exercise in the heat has been proposed to increase the risk for various heat-related illnesses with particular concerns regarding caffeine’s effect on body temperature and hydration status [357]. Ely et al. [358] concluded that caffeine dosages as high as 9 mg/kg did not substantially alter body heat balance during endurance exercise performance at 40 °C. Further, a recent study demonstrated that while caffeine ingestion increased blood lactate and heart rate during exercise in the heat (42 °C and 20% relative humidity), endurance capacity and thermoregulation were unaffected in both male and female participants [359]. Although caffeine may induce mild fluid loss, the majority of research has confirmed that caffeine consumption does not significantly impair hydration status, exacerbate dehydration, or jeopardize thermoregulation (i.e., body temperature regulation) when exercising in the heat [360, 361].

Several trials have observed no benefit of acute caffeine ingestion on cycling and running performance in the heat (Table 2) [265, 362, 364]. However, Ganio and colleagues [365] found caffeine (2 dosages of 3 mg/kg) to be similarly ergogenic under both cool (12 °C) and warm (33 °C) environmental conditions. Likewise, others [366] have reported a non-significant, yet notable, improvement in cycling time trial performance in the heat (35 °C and 25% relative humidity) after caffeine consumption (3 mg/kg) compared with placebo ingestion. While caffeine’s effect on performance in the heat remains somewhat unclear to date, positive support exists for dosages between 3 and 6 mg/kg. Further, there does not appear to be sufficient evidence to interdict the use of caffeine by individuals who exercise in heat if consumed in dosages of 9 mg/kg or less.

**Table 2:** Summary of studies that explored the effects of caffeine on exercise performance in the heat

Author	Participants	Protocol	Outcome
Cohen et al. 1996 [362]	Endurance trained competitive road racers (male = 5; female = 2)	• Placebo • **5 mg/kg caffeine** • **9 mg/kg caffeine**	⬌ Running performance
Del Coso et al. 2008 [363]	Endurance trained male cyclists (*n* = 7)	• No fluid • Water • 6%CHO Solution • **No fluid + 6 mg/kg caffeine capsule** • **Water + 6 mg/kg caffeine capsule** • **6%CHO solution + 6 mg/kg caffeine capsule**	⬌ Maximal voluntary contraction in heat *↑ Maximal cycling power in heat *↑ Maximal leg force via voluntary activation **ONLY in water + caffeine and 6%CHO + caffeine**
Cheuvront et al. 2009 [364]	Healthy males (*n* = 10)	• **9.0 mg/kg caffeine** • Placebo	⬌ TT performance ⬌ RPE
Ganio et al. 2011 [365]	Male cyclists (*n* = 11)	• Participants consumed either 3 mg/kg caffeine or placebo 60 min prior to and after 45 min of the following trials (4 trials total; total 6 mg/kg): • Warm environment (33 °C): 90 min cycling followed by 15 min performance trial • Cool environment (12 °C): 90 min cycling followed by 15 min performance trial	Caffeine *↑ increased performance versus placebo independent of temperature
Roelands et al. 2011 [265]	Trained male cyclists or triathletes (*n* = 8)	• **6 mg/kg caffeine** • Placebo	⬌ Acute cycling TT performance
Pitchford et al. 2014 [366]	Well-trained males (*n* = 9)	• **3 mg/kg caffeine** • Placebo	⬌cycling TT performance in heat
Suvi et al. 2017 [359]	Healthy males (*n* = 13) and females (*n* = 10)	• **6 mg/kg caffeine** • Placebo	⬌ Time to walking exhaustion
Beaumont et al. 2017 [310, 367]	Recreationally active males (*n* = 8)	• **6 mg/kg caffeine** • Placebo	*↑ Endurance cycle performance in heat *↓ RPE during initial 60 min of exercise

Outcomes are bold caffeine group specific; * = significant difference, ⬌ = no change, ↑ = improved performance, TT = time trial, mg/kg = milligram per kilogram, CHO = carbohydrate, min = minutes, RPE = rating of perceived exertion

#### Altitude

It is well established that caffeine improves performance and perceived exertion during exercise at sea level [260, 314, 368, 369]. Despite positive outcomes at sea level, minimal data exist on the ergogenic effects or side effects of caffeine in conditions of hypoxia, likely due to accessibility of this environment or the prohibitive costs of artificial methods. To date, only four investigations (Table 3) have examined the effects of caffeine on exercise performance under hypoxic conditions [211, 370–372]. In an initial study by Berglund and colleagues [370], caffeine (6 mg/kg) significantly improved 21 km time trial performance 2300 m above sea level in 14 well-trained cross-country skiers. Likewise, [371] positive outcomes were reported after caffeine (4 mg/kg) ingestion on endurance performance in acute hypoxic conditions of 4300 m above sea level. Specifically, significant improvements in time to exhaustion in eight young adults cycling at 80% of their altitude specific VO_2max_ was reported. More recently, 13 skiers were examined at an altitude of 2000 m above sea level and it was reported that caffeine (4.5 mg/kg) significantly improved time to exhaustion while double poling during cross-country skiing at 90% of altitude-specific VO_2max_ [211]. In a more recent double-blind, randomized, counterbalanced cross-over investigation [372], seven adult males significantly improved time to exhaustion by 12% following consumption of 4 mg/kg caffeine. Overall, results to date appear to support the beneficial effects of caffeine supplementation that may partly reduce the negative effects of hypoxia on the perception of effort and endurance performance [211, 370–372].

**Table 3:** Summary of studies that explored the effects of caffeine on exercise performance at altitude

Author	Participants	Protocol	Outcome
Berglund et al. 1982 [370]	Well-trained cross-country skiers (*n* = 14)	• **6 mg/kg caffeine** • Placebo	*↑ 21 km TT 2900 m above sea level
Fulco et al. 1994 [371]	Young adult cyclists (*n* = 8)	• **4 mg/kg caffeine** • Placebo	*↑ Time to exhaustion at 80% of their altitude-specific VO2max at 4300 m above sea level
Stadheim et al. 2015 [211]	Male sub-elite cross-country skiers (*n* = 13)	• **4.5 mg/kg caffeine** • Placebo	*↑ Double-poling time to task failure at 2000 m above sea level
Smirmaul et al. 2017 [372]	Adult male volunteers (*n* = 7)	• **4.0 mg/kg caffeine** • Placebo	*↑ Time to exhaustion during cycling by 12%

Outcomes are bold caffeine group specific; * = significant difference, ↑ = improved performance, TT = time trial, m = meters, mg/kg = milligram per kilogram

### Alternate caffeine sources

Sources other than commonly consumed coffee and caffeine tablets have garnered interest, including caffeinated chewing gum, mouth rinses, aerosols, inspired powders, energy bars, energy gels and chews, among others. While the pharmacokinetics [18, 373–376] and effects of caffeine on performance when consumed in a traditional manner, such as coffee [47, 49, 55, 153, 368, 377, 378] or as a caffeine capsule with fluid [55, 203, 379, 380] are well understood, curiosity in alternate forms of delivery (as outlined in pharmacokinetics section) have emerged due to interest in the speed of delivery [81]. A recent review by Wickham and Spriet [5] provides an overview of the literature pertaining to caffeine use in exercise, in alternate forms. Therefore, here we only briefly summarize the current research.

#### Caffeinated chewing gum

Several investigations have suggested that delivering caffeine in chewing gum form may speed the rate of caffeine delivery to the blood via absorption through the extremely vascular buccal cavity [58, 381]. Therefore, caffeine via chewing gum may be absorbed via two passageways: the buccal mucosa in the oral cavity and/or gut absorption due to the swallowing of caffeine-containing saliva [58, 381, 382]. Kamimori and colleagues [58] compared the rate of absorption and relative caffeine bioavailability from caffeinated chewing gum and caffeine in capsule form. The results suggest that the rate of drug absorption from the gum formulation was significantly faster. In the groups ingesting 100 and 200 mg, both gum and capsule formulations provide near comparable plasma caffeine concentrations to the systemic circulation. These findings suggest that there may be an earlier onset of pharmacological effects from caffeine delivered through the gum formulation. Further, while no data exist to date, it has been suggested that increasing absorption via the buccal cavity may be preferential over oral delivery if consumed closer to or during exercise, as splanchnic blood flow is often reduced [383], potentially slowing the rate of caffeine absorption.

To date, five studies [59–63] have examined the potential ergogenic impact of caffeinated chewing gum on aerobic performance, commonly administered in multiple sticks (Table 4). To note, all studies have been conducted using cycling interventions, with the majority conducted in well-trained cyclists. Results from these investigations suggest that caffeinated chewing gum delivered in total dosages ranging 200–300 mg, closer to initiation of exercise or during a prolonged endurance event may be most beneficial, specifically for individuals with a higher training status. However, more research is needed, especially in physically active and recreationally training individuals.

**Table 4:** Investigations examining the effects of caffeinated chewing gum on caffeine absorption and exercise performance

Author	Participants	Protocol	Results
Kamimori et al. 2002 [58]	Healthy males (*n* = 84; 12 per group)	• 50 mg caffeine capsule • 50 mg caffeine gum • 100 mg caffeine capsule • 100 mg caffeine gum • 200 mg caffeine capsule • 200 mg caffeine gum • Placebo	Both 100 and 200 mg of caffeine in gum and capsule formualtions provide comparable amounts of caffeine to the systemic circulation. Mean *T*_max_ for the gum groups ranged from 44.2 to 80.4 min as compared with 84.0–120.0 min for the capsule groups
Ryan et al. 2012 [59]	College-aged, physically active males (*n* = 8)	• **200 mg caffeinated gum** • Placebo gum	⬌ cycling TTE
Ryan et al. 2013 [60]	Well-trained male cyclists (*n* = 8)	• **300 mg caffeine gum** • Placebo gum	*↑ cycling TT performance when 300 mg caffeine chewing gum was administered 5 min pre-TT
Lane et al. 2014 [61]	Well-trained males (*n* = 12) and females (*n* = 12)	• **3 mg/kg caffeine gum 40 min prior + 1 mg/kg 10 min prior** • Placebo gum • Beet root juice • Beet root juice w/ caffeine	*↑ cycling TT performance by 3–4% Note: participant’s sex was accounted for during testing (female: 29.35 km; male: 43.83 km)
Oberlin-Brown et al. 2016 [62]	Well-trained male cyclists (*n* = 11)	• **200 mg caffeine gum** • **200 mg caffeine + CHO gum** • CHO gum • Placebo gum	⬌ cycling TT performance
Paton et al. 2015 [63]	Well-trained male (*n* = 10) and female (*n* = 10) cyclists	• **~ 3–4 mg/kg caffeine gum** • Placebo gum	~ 3–4 mg/kg enhanced both endurance (> 5 min) and sprint power output (< 30 s) by similar amounts (~ 4%) during the final 10 km of a 30-km race
Paton et al. 2010 [64]	Competitive male cyclists (*n* = 9)	• **3 mg/kg caffeine gum** • Placebo gum	*↓ power output decline in 3rd & 4th sprints
Bellar et al. 2012 [65]	Collegiate shot-put athletes (*n* = 9)	• **100 mg caffeine gum** • Placebo gum	*↑ shot-put performance
Ranchordas et al. 2018 [66]	Collegiate male soccer players (*n* = 10)	• **200 mg caffeine gum** • Placebo gum	*↑ Yo-Yo Intermittent Recovery Test level 1 and countermovement jump
Venier et al. 2019 [67, 68]	Resistance-trained men (*n* = 19)	• **300 mg caffeine gum** • Placebo gum	*↑ Jumping height *↑ Isokinetic strength and power *↑ Movement velocity in the bench press *↑ Whole-body power output

Bold text associated with reported trial outcomes; * delineates a significant change, NS = non-significant change, TT = Time Trial, TTE = time to exhaustion, ⬌ = no improvement/change, ↑ = improved performance, ↓ = decreased, min = minute, CHO = carbohydrate, seg = segment, *T*_max_ = maximum concentration, km = kilometers

Four studies [64, 66, 68, 384] have examined the effect of caffeinated chewing gum on more anaerobic type activities (Table 4). Specifically, Paton et al. [64] administered 3 mg/kg caffeinated gum to male cyclists during repeat sprint cycling, resulting in greater attenuation of fatigue, compared to a placebo. The reduced fatigue in the caffeine trials equated to a 5.4% performance enhancement in power during sprints, in favor of caffeinated gum. A study [384] assessing 100 mg caffeinated chewing gum on shot-put performance during an early morning trial resulted in overall improvements in shot-put distance thrown compared to a placebo. Caffeinated gum consumption also positively influenced performance in two out of three soccer-specific (Yo-Yo Intermittent Recovery Test and CMJ) tests used in the assessment of performance in soccer players [66]. A recent study also explored the effects of 300 mg of caffeine provided in caffeine chewing gum and found that its consumption 10 min pre-exercise resulted in ergogenic effects on jumping performance, isokinetic peak torque, upper body movement velocity and whole-body power output during a rowing test [68]. These results suggest that caffeine chewing gums may provide ergogenic effects across a wide range of exercise tasks. To date, only Bellar et al. [384] has examined chewing gum with caffeine on cognitive function, specifically reporting improved alertness as assessed by a psychomotor vigilance test. Future studies may consider comparing the effects of caffeine in chewing gums to caffeine ingested in capsules.

#### Caffeine mouth rinsing

Caffeine mouth rinsing (CMR; 5–20 s in duration) may have the potential to enhance exercise performance due to the activation of sensorimotor brain cortices [79]. Specifically, the mouth contains bitter taste sensory receptors that are sensitive to caffeine [385]. It has been proposed that activation of these bitter taste receptors may activate neural pathways associated with information processing and reward within the brain [385–387]. Physiologically, caffeinated mouth rinsing may also reduce gastrointestinal distress potential that may be caused when ingesting caffeine sources [388, 389].

Few investigations on aerobic [69, 74–76, 390] and anaerobic [72, 73, 78] changes in performance, as well as cognitive function [70, 71] and performance [77], following CMR have been conducted to date (Table 5). One study [390] demonstrated ergogenic benefits of CMR on aerobic performance, reporting significant increases in distance covered during a 30-min arm crank time trial performance. Likewise, in a separate study [74], a 5 s CMR (containing 32 mg of caffeine dissolved in 125 ml water) improved 30 min cycling performance, without concurrent increases in ratings of perceived exertion or heart rate. With regard to anaerobic trials, other researchers [72] have also observed improved performance, where recreationally active males significantly improved their mean power output during repeated 6-s sprints after rinsing with a 1.2% caffeine solution. A follow-up study [73] reported that recreationally active males who were deemed ‘glycogen depleted’ increased mean and peak power during the 3rd sprint of repeat cycling, as well as decreased perception of pain during the 4th and 5th sprints following a 10 s rinse of a 2% caffeine rinse. While CMR has demonstrated positive outcomes for cyclists, another study [78] in recreationally resistance-trained males did not report any significant differences in the total weight lifted by following a 1.2% caffeine rinse. CMR appears to be ergogenic in cycling to include both longer, lower-intensity and shorter high-intensity protocols. The findings on the topic are equivocal likely because caffeine provided in this source does not increase caffeine plasma concentration and increases in plasma concentration are likely needed to experience an ergogenic effect of caffeine [69]. Details of these studies, as well as additional studies may be found in Table 5.

**Table 5:** Investigations examining the effects of caffeine mouth rinsing (CMR) on exercise performance

Author	Participants	Protocol	Results
Doering et al. 2014 [69]	Well-trained cyclists (*n* = 10)	• **10 s rinse 35 mg caffeine/25 mL X 8** • Placebo rinse	⬌ plasma caffeine levels ⬌ cycling TT Performance
De Pauw et al. 2015 [70]	Healthy males (*n* = 10)	• **20 s- 25 mL Rinse 1.2% caffeine** • 20 s- 25 mL Rinse 6.4% CHO • Placebo Rinse	*↑ stroop task performance
Pomportes et al. 2017 [71]	Physically active males (*n* = 16) and females (*n* = 6)	• **20 s- 25 mL rinse 67 mg caffeine** • **20 s- 25 mL rinse 7.0% CHO** • **20 s- 25 mL rinse 0.4 g guarana** • Placebo rinse	⬌ variability or production durations ⬌ errors made
Beaven et al. 2013 [72]	Recreationally active males (*n* = 12)	• **5 s- 25 mL rinse 1.2% caffeine** • 5 s- 25 mL rinse 6% CHO • Placebo rinse	*↑ mean power in first sprint for caffeine and CHO rinses NS ↑ maximal power in first two sprints
Beaven et al. 2013 [72]	Recreationally active males (*n* = 12)	• 5 s- 25 mL rinse 1.2% caffeine • 5 s- 25 mL rinse 6.0% CHO • **5 s- 25 mL rinse 1.2% caffeine** **+ 6.0% CHO**	*↑ peak power in first sprint *↑ mean power in fifth sprint
Kizzi et al. 2016 [73]	Glycogen depleted, recreationally active males (*n* = 8)	• **10 s- 25 mL rinse 2.0% caffeine** • Placebo rinse	⬌ mean and peak power in 4th and 5th sprint
Sinclair and Bottoms 2014 [74]	Healthy males (*n* = 12)	• **5 s- 25 mL rinse 0.032% caffeine** • **5 s- 25 mL rinse 6.4% CHO** • Placebo rinse	*↑ arm crank TT performance
Bottoms et al. 2014 [74]	Healthy males (*n* = 12)	• **5 s- 125 mL rinse w/ 32 mg of caffeine** • 5 s – 6.4% CHO solution • Placebo rinse	*↑ distance cycled during the caffeine mouth rinse trial (16.2 ± 2.8 km) was significantly greater compared to placebo trial (14.9 ± 2.6 km). There was no difference between CHO and caffeine trials
Pataky et al. 2016 [75]	Recreationally trained male (*n* = 25) and female (*n* = 13) cyclists	• **Placebo rinse + 6 mg/kg caffeine capsule** • 25 mL rinse 300 mg caffeine + placebo capsule • **25 mL rinse 300 mg caffeine + 6 mg/kg caffeine capsule**	*↑ 3 km cycling TT performance
Lesniak et al. 2016 [76]	Recreationally active females (*n* = 7)	• 5 s- 25 mL rinse 1.2% caffeine • 5 s- 25 mL rinse 6.0% CHO • 5 s- 25 mL rinse 1.2% caffeine + 6% CHO	⬌ cycling TT performance
Dolan et al. 2017 [77]	College lacrosse players (*n* = 10)	• 5 s- 25 mL Rinse 1.2% caffeine • 5 s- 25 mL Rinse 6.0% CHO • 5 s- 25 mL Rinse 1.2% caffeine + 6.0% CHO • Placebo rinse • No rinse	⬌ intermittent sport performance
Clarke et al. 2015 [78]	Recreationally resistance-trained males (*n* = 15)	• 5 s- 25 mL rinse 1.2% caffeine • 5 s- 25 mL rinse 6.0% CHO • 5 s- 25 mL rinse 1.2% caffeine + 6.0% CHO • Placebo rinse	⬌ total weight lifted

Bold text associated with reported trial outcomes; s = seconds, mL = milliliters, CHO = carbohydrate, TT time trial, * = significant difference, NS = non-significant difference, ↑ = improved performance, ↓ = decreased, ⬌ = no improvement/change, mg = milligrams

#### Caffeinated nasal sprays and inspired powders

The use of caffeinated nasal sprays and inspired powders are also of interest. Three mechanisms of action have been hypothesized for caffeinated nasal sprays. Firstly, the nasal mucosa is permeable, making the nasal cavity a potential route for local and systemic substance delivery; particularly for caffeine, a small molecular compound [11, 12, 30, 31]. Secondly, and similar to CMR, bitter taste receptors are located in the nasal cavity. The use of a nasal spray may allow for the upregulation of brain activity associated with reward and information processing [391]. Thirdly, but often questioned due to its unknown time-course of action, caffeine could potentially be transported directly from the nasal cavity to the CNS, specifically the cerebrospinal fluid and brain by intracellular axonal transport through two specific neural pathways, the olfactory and trigeminal [392, 393].

In two separate trials [79, 80], the effects of caffeinated nasal sprays containing 15 mg of caffeine per mL were examined. No significant improvements were reported in either anaerobic and aerobic performance outcome measures despite the increased activity of cingulate, insular, and sensory-motor cortices [79]. Laizure et al. [81] compared the bioavailability and plasma concentrations of 100 mg caffeine delivered via an inspired powder (AeroShot) and an oral solution. Both were found to have similar bioavailability and comparable plasma concentrations with no differences in heart rate or blood pressure (Table 6).

**Table 6:** Investigations examining the effects of caffeine nasal sprays on exercise and cognitive performance

Author	Participants	Protocol	Results
De Pauw et al. 2017 [79]	Healthy males (*n* = 10)	• **Nasal spray 15 mg/mL caffeine** • Nasal spray 80 mg/mL glucose • Placebo nasal spray	*↑ activity of cingulate, insular, and sensory-motor cortices ⬌ stroop task performance
De Pauw et al. 2017 [80]	Moderately trained males (*n* = 11)	• Nasal spray 15 mg/mL caffeine • Nasal spray 80 mg/mL glucose • Placebo nasal spray	⬌ plasma caffeine levels ⬌ wingate performance ⬌ 30 min cycling TT performance ⬌ stroop task performance
Laizure et al. 2017 [81]	Healthy adults (*n* = 17)	• Inspired powder 100 mg/mL caffeine (AeroShot) • Oral solution 100 mg/248 mL caffeine	⬌ peak plasma caffeine levels ⬌ bioavailability

mg/mL = milligram per milliliter, TT = time trial, * = significant difference, ⬌ = no improvement/change, ↑ = increased, ↓ = decreased, min = minute

#### Caffeinated gels

While caffeinated gels are frequently consumed by runners, cyclists and triathletes, plasma caffeine concentration studies have yet to be conducted and only three experimental trials have been reported. Cooper et al. [83] and Scott et al. [84] examined the effects of carbohydrate-caffeinated gels, which both included 100 mg caffeine dosages alongside 25 g and 21.6 carbohydrate, respectively. In the study by Cooper et al. the consumption of caffeinated gels 60 min pre-exercise did not enhance intermittent sprint performance. In contrast, Scott et al. utilized a shorter time period from consumption to the start of the exercise (i.e., 10 min pre-exercise) and found significant improvements in 2000-m rowing performance after consumption of caffeinated gels. Another recent study utilized caffeine gels and found that 300 mg of caffeine, provided 10 min pre-exercise increased vertical jump performance, strength, and power in a sample of 17 resistance-trained men [67]. These results tentatively suggest that timing of consumption is important when it comes to caffeinated gels with ergogenic effects found when consuming caffeine gels 10 min but not 60 min before exercise. However, these ideas are based on results from independent studies and therefore, future studies may consider exploring the optimal timing of caffeine gel ingestion in the same group of participants. More details on these studies may be found in (Table 7).

**Table 7:** Investigations examining the effects of caffeinated bars and gels on exercise performance

Author	Participants	Protocol	Results
Hogervorst et al. 2008 [82]	Well-trained male cyclists (*n* = 24)	• **Bar with 100 mg caffeine and 45.0 g CHO** • Bar with only 45.0 g CHO • 300 mL non-caloric beverage	*↑Stroop and Rapid Visual Information Processing tests after 140 min and time to exhaustion exercise trial at 75% VO2_max_
Cooper et al. 2014 [83]	Recreationally trained males (*n* = 12)	• **Gel with 100 mg caffeine and 25.0 g CHO** • Gel with 25 g CHO • Gel placebo	*↓ fatigue and RPE during 3rd sprint set NS: sprint performance
Scott et al. 2015 [84]	Male college athletes (*n* = 13)	• **Gel with 21.6 g CHO and 100 mg caffeine** • Gel with 21.6 g CHO	*↑ performance in 2000 m rowing task
Venier et al. 2019 [67, 68]	Resistance-trained men (*n* = 17)	• **Gel with 88 g CHO and 300 mg caffeine** • Gel with 88 g CHO	*↑ jumping height *↑ isokinetic strength and power *↑ movement velocity in the bench press NS: whole-body power output

Bold text associated with reported trial outcomes; mg = milligrams, g = grams, CHO = carbohydrate, * = significant, NS = non-significant difference, VST = visual sensitivity test, ↑ = improved performance, ↓ = decreased, m = meters, RPE = rating of perceived exertion, mL = milliliters

#### Caffeinated bars

Similar to caffeinated gels, no studies measured plasma caffeine concentration following caffeinated bar consumption; however, absorption and delivery likely mimic that of coffee or caffeine anhydrous capsule consumption. While caffeinated bars are commonly found in the market, research on caffeinated bars is scarce. To date, only one study [82] (Table 7) has examined the effects of a caffeine bar on exercise performance. Specifically, participants that consumed a carbohydrate-bar containing 100 mg of caffeine significantly improved their time to exhaustion during cycling compared to a carbohydrate bar and placebo with no differences found in ratings of perceived exertion, average heart rate and relative exercise intensity. Furthermore, cyclists significantly performed better on complex information processing tests following the time trial to exhaustion after caffeine bar consumption when compared to the carbohydrate only trial. As there is not much data to draw from, future work on this source of caffeine is needed.

### Caffeine in combination with other ingredients

#### Caffeine and Creatine

A review by Trexler and Smith-Ryan comprehensively details research on caffeine and creatine co-ingestion [32]. With evidence to support the ergogenic benefits of both creatine and caffeine supplementation on human performance—via independent mechanisms—interest in concurrent ingestion is of great relevance for many athletes and exercising individuals [32]. While creatine and caffeine exist as independent supplements, a myriad of multi-ingredient supplements (e.g., pre-workouts) containing both caffeine and creatine are available. It has been reported that the often-positive ergogenic effect of acute caffeine ingestion prior to exercise is unaffected by creatine when a prior creatine loading protocol had been completed by participants [394, 395]. However, there is some ambiguity with regard to the co-ingestion of caffeine during a creatine-loading phase (e.g., co-ingestion of coffee and creatine) [396–399]. Studies available to date suggest that high-dose chronic caffeine (> 9 mg/kg) and creatine co-ingestion should be employed cautiously, as counteracting mechanisms on Ca2+ clearance and release, and muscle relaxation time have been hypothesized [396, 398]. While favorable data exist on muscular performance outcomes and adaptations in individuals utilizing multi-ingredient supplements (e.g., pre-workouts), these results may be confounded by other ingredients (e.g., beta-alanine, citrulline malate, amino acids) in the supplement [34, 95, 400, 401]. Until future investigations are available, it may be prudent to consume caffeine and creatine separately, or avoid high caffeine intakes when utilizing creatine for muscular benefits [402].

#### Caffeine and carbohydrate

To date, investigations examining the co-ingestion of carbohydrate and caffeine compared with carbohydrate alone prior to and/or during exercise have produced inconsistent results [196, 264, 403–405]. This is likely due to the heterogeneity of experimental protocols that have been implemented and examined. Nonetheless, a 2011 systematic review and meta-analysis of 21 investigations [406] concluded the co-ingestion of carbohydrate and caffeine significantly improved endurance performance when compared to carbohydrate alone. However, it should be noted that the magnitude of the performance benefit that caffeine provides is less when added to carbohydrate (i.e., caffeine + carbohydrate vs. carbohydrate) than when isolated caffeine ingestion is compared to placebo [404]. Since the 2011 publication [406], results remain inconclusive, as investigations related to sport-type performance measures [83, 250, 407–411], as well as endurance performance [84, 367, 412] continue to be published. Overall, to date it appears caffeine alone, or in conjunction with carbohydrate is a superior choice for improving performance, when compared to carbohydrate supplementation alone.

While the majority of training or performing individuals would choose to supplement with caffeine prior to exercise or during competition, interest in caffeine’s effect on muscle glycogen repletion during the post-exercise period has garnered interest. Few studies to date have investigated the effect of post-exercise caffeine consumption on glucose metabolism [413, 414]. While the delivery of exogenous carbohydrate can increase muscle glycogen alone, Pedersen et al. [413] report faster glycogen repletion rates in athletes who co-ingested caffeine (8 mg/kg body mass) and carbohydrate (4 g/kg body mass), compared to carbohydrate alone (4 g/kg body mass). In addition, it has been demonstrated that co-ingestion of caffeine with carbohydrate after exercise improved subsequent high-intensity interval-running capacity compared with ingestion of carbohydrate alone. This effect may be due to a high rate of post-exercise muscle glycogen resynthesis [415]. The data to date indicate that caffeine may potentiate glycogen resynthesis when high dosages of caffeine (~ 8 mg/kg body mass) are consumed during the recovery phase of exercise; though, when adequate carbohydrate is provided post-exercise, caffeine may not provide any glycogen-resynthesizing benefit [414]. Practically, caffeine ingestion in close proximity to sleep, coupled with the necessity to speed glycogen resynthesis, should be taken into consideration, as caffeine before bed may cause sleep disturbances.

#### Caffeine within brewed coffee

The genus of coffee is *Coffea*, with the two most common species *Coffea arabica* (arabica coffee) and *Coffea canephora* (robusta coffee) used for global coffee production. While coffee is commonly ingested by exercising individuals as part of their habitual diet, coffee is also commonly consumed pre-exercise to improve energy levels, mood, and exercise performance [11, 40]. Indeed, a recent review on coffee and endurance performance, reported that that coffee providing between 3 and 8.1 m/kg of caffeine may benefit endurance performance, such as time trial performance or time to exhaustion [11]. To date, research has only examined coffee’s effects on cycling and running exercise performance. Specifically, Higgins et al. [11] highlight that significant improvements over control conditions were found with doses up to 8.1 mg/kg; however, performance benefits were similar to 3 mg/kg servings. Since the release of the Higgins et al. review, three additional studies have been published, examining the effects of coffee on exercise performance. Specifically, Niemen et al. [416] assessed the impact of high chlorogenic acid coffee on performance. Cyclists were asked to consume coffee or placebo (300 ml/day) for 2 weeks prior to completing a 50-km time-trial. Chlorogenic acid coffee provided 1066 mg chlorogenic acid plus 474 mg caffeine, while the placebo consisted of 187 mg chlorogenic acid and 33 mg caffeine. Fifty-km cycling time performance and power did not differ between trials. Participant’s heart rate and ventilation were higher with chlorogenic acid coffee during the time-trial, potentially provoking the non-significant performance outcomes. Regarding resistance exercise performance, only two studies [55, 56] have been conducted to date. One study [56] reported that coffee and caffeine anhydrous did not improve strength outcomes more than placebo supplementation. On the other hand, Richardson et al. [55] suggested that coffee consumption may improve lower-body muscular endurance performance similarly as isolated caffeine ingestion. The results between studies differ likely because it is challenging to standardize the dose of caffeine in coffee as differences in coffee type and brewing method may alter caffeine content [417]. Even though coffee may enhance performance, due to the difficulty of standardizing caffeine content most sport dietitians and nutritionists use anhydrous caffeine with their athletes due to the difficulty of standardizing caffeine content.

#### Caffeine containing energy drinks and pre-workouts

Consumption of energy drinks has become more common in the last decade, and several studies have examined the effectiveness of energy drinks as ergogenic aids (Table 8). Souza and colleagues [418] completed a systematic review and meta-analysis of published studies that examined energy drink intake and physical performance. Studies including endurance exercise, muscular strength and endurance, sprinting and jumping, as well as sport-type activities were reviewed. Dosages of caffeine ranged from 40 to 325 mg among the studies, with the majority of drinks also containing taurine. While it was concluded that energy drink consumption increased performance in the aforementioned performance activities, the ergogenic effect was not solely attributed to the amount of caffeine administered, but improved also as a result of taurine content (dosages ranged 71 to 3105 mg) [418]. This is similar to data from another study, reporting that Red Bull (500 ml serving; 160 mg of caffeine/2.25 mg/kg), also containing taurine, glucose, glucuronolactone, and B vitamins, improved 5-km run performance in recreationally athletes [91]. It has been suggested that the additional taurine to caffeine containing energy drinks or pre-workout supplements, as well as the addition of other ergogenic supplements such as beta-alanine, B-vitamins, and citrulline, may potentiate the effectiveness of caffeine containing beverages on athletic performance endeavors [419]. However, other suggest that the ergogenic benefits of caffeine containing energy drinks is likely attributed to the caffeine content of the beverage [420]. For a thorough review of energy drinks, consider Campbell et al. [419]. Table 8 provides a review of research related to energy drinks and pre-workout supplements.

**Table 8:** Energy drinks and pre-workout supplements

	Author	Participants	Protocol	Results	Other supplements
Endurance Exercise Performance	Alford et al. 2001 [85]	Young adults (*n* = 36)	**-250 mL Energy drink with 80 mg caffeine and 26 g CHO** -Carbonated placebo -No drink	*↑ Aerobic Endurance	**-26 g CHO**
	Candow et al. 2009 [86]	Young men (*n* = 9) and women (*n* = 8)	**-CHO free energy drink with 2 mg/kg caffeine** -Non-caffeinated version of energy drink	⬌ High-intensity Run Time to Exhaustion	
	Walsh et al. 2010 [87]	Recreationally active men (*n* = 9) and women (*n* = 6)	**-26 g Pre-workout with unknown amount of caffeine (ingredients listed in column 5)** -Placebo	*↑ Mod-intensity Run Time to Exhaustion	**−2.05 g taurine, caffeine, and gluconolactone, 7.9 g L-leucine, L-isoleucine, L-valine, L-arginine and L-glutamine, 5 g of di-creatine citrate, and 2.5 g of βalanine**
	Ivy et al. 2009 [88]	Trained cyclists men (*n* = 6) and women (*n* = 6)	**-Energy drink with 160 mg caffeine** -Placebo	*↑ Cycle Time Trial Performance by 4.7%	**-2.0 g taurine, 1.2 g glucuronolactone, 54 g carbohydrate, 40 mg niacin, 10 mg pantothenic acid, 10 mg vitamin B6, and 10 microg vitamin B12**
	Sanders et al. 2015 [89]	Healthy participants (*n* = 15)	−12 oz. Placebo (Squirt) **− 8.4 oz. Red Bull®** **− 16 oz. Monster Energy®** **− 2 oz. 5-h ENERGY®**	⬌ RPE on Treadmill at 70% VO_2_ max ⬌ Oxygen Consumption at 70% VO_2_ max	
	Al-Fares et al. 2015 [90]	Healthy female students (*n* = 32)	**-Energy drink with 160 mg caffeine** -Placebo with similar CHO content	⬌ VO_2_ max	**−2.0 g taurine, 1.2 g glucuronolactone, 54 g carbohydrate, 40 mg niacin, 10 mg pantothenic acid, 10 mg vitamin B6, and 10** μg **vitamin B12**
	Prins et al. 2016 [91]	Recreation endurance male (*n* = 13) and female (*n* = 5) runners	**-Energy drink with 160 mg caffeine** -Placebo	*↑ 5 k Time Trial	**−2.0 g taurine, 1.2 g glucuronolactone, 54 g carbohydrate, 40 mg niacin, 10 mg pantothenic acid, 10 mg vitamin B6, and 10 microg vitamin B12**
	Kinsinger et al. 2016 [92]	Recreational male athletes (*n* = 23)	**−1.93 oz Energy shot with 100 mg caffeine** − 1.93 oz. Placebo	⬌ RPE on Treadmill VO_2_ max Test ⬌ Treadmill VO_2_ max	**-1870 mg (taurine, glucuronic acid, malic acid, N-acetyl L-tyrosine, L-phenylalanine and citicoline)**
Resistance/Sprint Performance	Forbes et al. 2007 [93]	Young men (*n* = 11) and women (*n* = 40	**-Energy drink with 2 mg/kg caffeine** -Non-caffeinated version of energy drink	*↑ Bench-Press Repetitions by 6%	
	Del Coso et al. 2012 [94]	Healthy men (*n* = 9) and women (*n* = 3)	-Energy drink with 1 mg/kg caffeine **-Energy drink with 3 mg/kg caffeine** -Non-caffeinated version of energy drink	*↑ Half-Squat Maximal Power by 7% *↑ Bench-Press Maximal Power by 7%	
	Gonzalez et al. 2011 [95]	Resistance-trained college males (*n* = 8)	**−26 g Pre-workout with unknown amount of caffeine (ingredients listed in column 5)** -Placebo	*↑ # of Bench-Press and Squat Repetitions at 80% 1RM by 11.8% *↑ Average Power Output for the Workout	**-2.05 g taurine, caffeine, and gluconolactone, 7.9 g L-leucine, L-isoleucine, L-valine, L-arginine and L-glutamine, 5 g of di-creatine citrate, and 2.5 g of βalanine**
	Astorino et al. 2011 [96]	Collegiate female soccer players (*n* = 15)	**-255 mL energy drink with 1.3 mg/kg caffeine + 1 g taurine** -Placebo	⬌ Sprint-based Exercise Performance	**-1 g taurine**
	Campbell et al. 2016 [97]	College men (*n* = 8) and women (*n* = 11)	**-37 mL Energy shot with 2.4 mg/kg caffeine** -37 mL Placebo	⬌ Vertical Jump ⬌ YMCA Bench-Press NS↑ Curl-up Endurance	
	Eckerson et al. 2013 [98]	Resistance-trained men (*n* = 17)	**−500 mL Energy drink with 160 mg caffeine + 2 g taurine** − 500 mL Energy drink with 160 mg caffeine − 500 mL Placebo	⬌ 1RM Bench-Press Strength ⬌ Total Volume Lifted	**−2 g Taurine**
	Astley et al. 2018 [99]	Resistance-trained men (*n* = 15)	**-Energy drink with 2.5 mg/kg caffeine** -Non-caffeinated version of energy drink	*↑ Knee Extensions in Dominant Leg *↑ 80% 1RM Bench-Press Reps *↑ Isometric Grip Strength	
	Magrini et al. 2016 [100]	Healthy men (*n* = 23) and women (*n* = 8)	**-4 oz Energy drink with 158 mg caffeine** -4 oz. Placebo	⬌ Total Push-ups	
Anaerobic Exercise Performance for Power	Forbes et al. 2007 [93]	Young men (*n* = 11) and women (*n* = 4)	**-Energy drink with 2 mg/kg caffeine** -Non-caffeinated version of energy drink	⬌ Wingate Peak Power ⬌ Wingate Average Power	
	Campbell et al. 2010 [101]	Recreationally active young men (*n* = 9) and women (*n* = 6)	**-Energy drink with 2.1 mg/kg caffeine** -Non-caffeinated version of energy drink	⬌ Wingate Peak Power	
	Hoffman et al. 2009 [102]	Male strength/power athletes (*n* = 12)	**-Energy drink with 1.8 mg/kg caffeine** -Non-caffeinated version of energy drink	⬌ Wingate Power Performance	
	Alford et al. 2001 [85]	Young adults (*n* = 36)	**−250 mL Energy drink with 80 mg caffeine and 26 g CHO** -Carbonated placebo -No drink	*↑ Maximum Speed on Cycle Ergometer	**-26 g CHO**
	Campbell et al. 2016 [97]	College men (*n* = 8) and women (*n* = 11)	**-37 mL Energy shot with 2.4 mg/kg caffeine** -37 mL Placebo	⬌ Repeated Sprint Speed	
Mood/ Reaction Time/ Alertness	Alford et al. 2001 [85]	Young adults (*n* = 36)	**-250 mL Energy drink with 80 mg caffeine and 26 g CHO** -Carbonated placebo -No drink	*↑ Choice Reaction Time *↑ Concentration *↑ Memory	**-26 g CHO**
	Walsh et al. 2010 [87]	Recreationally active men (*n* = 9) and women (*n* = 6)	**-26 g Pre-workout with unknown amount of caffeine (ingredients listed in column 5)** -Placebo	*↑ Focus and Energy in 1st 10 min of Exercise ⬌ Energy, Fatigue, and Focus Immediately Post-exercise	**-2.05 g taurine, caffeine, and gluconolactone, 7.9 g L-leucine, L-isoleucine, L-valine, L-arginine and L-glutamine, 5 g of di-creatine citrate, and 2.5 g of βalanine**
	Hoffman et al. 2009 [102]	Male strength/power athletes (*n* = 12)	-**Energy drink with 1.8 mg/kg caffeine** -Non-caffeinated version of energy drink	*↓ Reaction Time *↑ Feelings of Energy and Focus NS↑ Alertness	
	Seidl et al. 2000 [103]	Male (*n* = 4) and female (*n* = 6) graduate students	**-Energy drink with 160 mg caffeine** -Placebo	*↓ Reaction Time at Night ⬌ Vitality Scores at Night [[when compared to the Placebo Group who saw a significant decline in vitality and response time]]	**2.0 g taurine, 1.2 g glucuronolactone, 54 g carbohydrate, 40 mg niacin, 10 mg pantothenic acid, 10 mg vitamin B6, and 10 microg vitamin B12**
	Scholey et al. 2004 [104]	Healthy volunteers (*n* = 20)	**-250 ml Energy drink with 75 mg caffeine** -Non-caffeinated version of energy drink -Placebo	*↑ Secondary Memory *↑ Speed of Attention	**−37.5 g glucose, ginseng, and** ***Ginkgo biloba***
	Smit et al. 2004 [105]	Healthy volunteers (*n* = 271)	-**Caffeine + CHO + Carbonation** -Placebo with carbonation -Placebo without carbonation	⬌ Mood and Performance (during fatiguing and cognitively demanding tasks)	**-CHO**
	Rao et al. 2005 [106]	Healthy volunteers (*n* = 40)	-**Caffeine + CHO** -Placebo	*↑ Event Related Potentials in ECGs *↑ Behavioral Performance in Accuracy and Speed of Performance	**-CHO**
	Howard et al. 2010 [107]	College students (*n* = 80)	-Energy drink with 1.8 ml/kg caffeine** **-Energy drink with 3.6 ml/kg caffeine** **-Energy drink with 5.4 ml/kg caffeine** -Non-caffeinated version of energy drink -No drink	Compared with the placebo and no drink conditions, the energy drink doses decreased reaction times on the behavioral control task, increased subjective ratings of stimulation and decreased ratings of mental fatigue. Greatest improvements in reaction times and subjective measures were observed with the lowest dose (1/8 mg/kg).	**Taurine, sucrose and glucose, B-group vitamins**
	Wesnes et al. 2016 [108]	Young volunteers (*n* = 24)	**-250 mL Energy drink with 80 mg caffeine + 27 g glucose** -250 mL Energy drink with 80 mg caffeine -250 mL Placebo	*↑ Working and Episodic Memory	**-27 g Glucose**

Outcomes are bold group specific; * = significant difference, ⬌ = no change, ↑ = improved performance, ↓ = decrease, TT = time trial, RPA = rating of perceived exertion, NS = non-significant improvement, mg/kg = milligram per kilogram, g = grams, CHO = carbohydrate, min = minutes, VO_2_ = aerobic capacity, m = meters, ml = milliliters, RPE = rating of perceived exertion

### Summary

Caffeine in its many forms is a ubiquitous substance frequently used in military, athletic and fitness populations which acutely enhance many aspects of exercise performance in most, but not all studies.

Supplementation with caffeine has been shown to acutely enhance many aspects of exercise, including prolonged aerobic-type activities and brief duration, high-intensity exercise. Caffeine is ergogenic when consumed in doses of 3–6 mg/kg body mass. The most commonly used timing of caffeine supplementation is 60 min pre-exercise. The optimal timing of caffeine ingestion likely depends on the source of caffeine. Caffeine’s effects seem to be similar in both trained and untrained individuals. Studies that present individual participant data commonly report substantial variation in caffeine ingestion responses. Inter-individual differences may be associated with habitual caffeine intake, genetic variations, and supplementation protocols in a given study. Caffeine may be ergogenic for cognitive function, including attention and vigilance. Caffeine may improve cognitive and physical performance in some individuals under conditions of sleep deprivation. Caffeine at the recommended doses does not appear significantly influence hydration, and the use of caffeine in conjunction with exercise in the heat and at altitude is also well supported. Alternative sources of caffeine, such as caffeinated chewing gum, mouth rinses, and energy gels, have also been shown to improve performance. Energy drinks and pre-workouts containing caffeine have been demonstrated to enhance both anaerobic and aerobic performance. Individuals should also be aware of the side-effects associated with caffeine ingestion, such as sleep disturbance and anxiety, which are often linearly dose-dependent.

## Data Availability

Not applicable.
